# On scattered waves and lipid domains: detecting membrane rafts with X-rays and neutrons

**DOI:** 10.1039/c5sm01807b

**Published:** 2015-10-02

**Authors:** Drew Marquardt, Frederick A. Heberle, Jonathan D. Nickels, Georg Pabst, John Katsaras

**Affiliations:** a University of Graz , Institute of Molecular Biosciences , Biophysics Division , NAWI Graz , Humboldtstr. 50/III , Graz , Austria . Email: georg.pabst@uni-graz.at ; Tel: +43 316 380 4989; b BioTechMed-Graz , Graz , Austria; c Oak Ridge National Laboratory , Oak Ridge , Tennessee 37831 , USA . Email: katsarasj@ornl.gov ; Tel: +1 865 274 8824; d Joint Institute for Neutron Sciences , Oak Ridge , Tennessee 37831 , USA; e Department of Physics , University of Tennessee , Knoxville , Tennessee 37996 , USA; f Department of Physics , Brock University , St. Catharines , Ontario L2S 3A1 , Canada; g Bredesen Center for Interdisciplinary Research and Graduate Education , University of Tennessee , Knoxville , Tennessee 37996 , USA

## Abstract

In order to understand the biological role of lipids in cell membranes, it is necessary to determine the mesoscopic structure of well-defined model membrane systems. Neutron and X-ray scattering are non-invasive, probe-free techniques that have been used extensively in such systems to probe length scales ranging from angstroms to microns, and dynamics occurring over picosecond to millisecond time scales. Recent developments in the area of phase separated lipid systems mimicking membrane rafts will be presented, and the underlying concepts of the different scattering techniques used to study them will be discussed in detail.

## Abstract 2

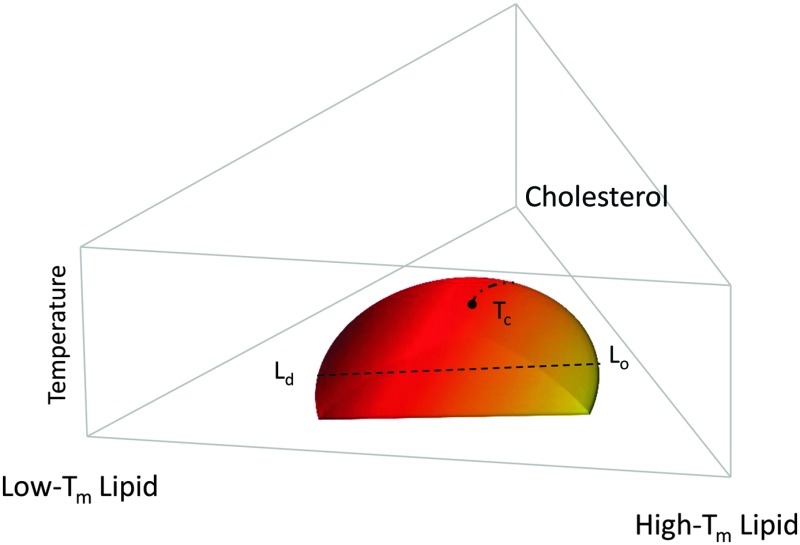
In order to understand the biological role of lipids in cell membranes, it is necessary to determine the mesoscopic structure of well-defined model membrane systems.

## 1 Introduction

Biological membranes are complex, self-assembled composites of proteins, lipids and carbohydrates, whose hierarchical organization is fundamental to physiological processes. In particular, lateral organization of the lipid/protein layer of plasma membranes has not only attracted significant scientific interest, but also considerable controversy. The membrane raft paradigm invokes the existence of functional domains enriched in sphingolipids, cholesterol and proteins, such as glycophosphatidylinositol-anchored proteins that facilitate diverse cellular signaling and transport processes.^1^ However, proof of their existence in live cells has been elusive.^2–4^

In contrast, domains are well-established in lipid-only model systems of plasma membranes.^5,6^ Such systems of reduced complexity allow for close scrutiny of the biophysical nature of lipid–lipid interactions and their potential in organizing lateral membrane structure. Over the years, a variety of experimental techniques have been applied to study the properties of lipid domains.^7^ In this tutorial review we focus on the ability of X-rays and neutrons to interrogate the properties of lipid domains, using either elastic or inelastic scattering. The present work can be seen as a follow-up to one of our previous review articles,^8^ which while briefly summarizing early scattering studies on lipid domains, was mainly focused on homogeneous lipid bilayers. Here we discuss progress in the field that has taken place over the past five years.

The review article is organized as follows. First, we give a brief introduction to lipid-only domains in model systems mimicking the plasma membrane. We then expand on the theory of elastic and inelastic scattering of lipid domains and describe some illustrative examples. Finally, we conclude and give an outlook as to what can be expected in this area of research in the near future.

## 2 Properties of membrane domains

Lipids in multi-component mixtures minimize free energies arising from their chemical structure, leading to differences in membrane structure, hydrocarbon chain packing and chain order, and hydrogen bond formation. For example, in a binary mixture of lipids (*e.g.*, A and B), these interactions can be parameterized by^9–11^
1
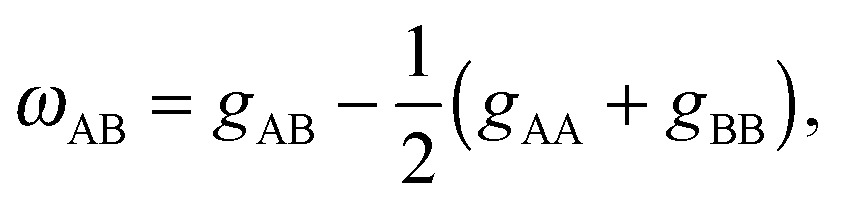
where *g*_AA_, *g*_BB_ and *g*_AB_ are the interaction free energies between like (AA and BB) and unlike (AB) pairs. Typical values for *ω*_AB_ vary between –1*k*_B_ *T* and +0.7*k*_B_ *T*,^12^ where phase separation occurs for *ω*_AB_ > +0.55*k*_B_ *T*, and random mixing for *ω*_AB_ = 0.^13^ Qualitatively, lipids prone to form gel phases (those with saturated acyl chains) and lipids prone to form fluid phases (unsaturated lipid species) will phase separate over a broad range of temperatures and compositions (reviewed by Marsh^6,14^).

When discussing lateral membrane heterogeneity, it is useful to distinguish between four cases: (i) random (ideal) mixing; (ii) non-random mixing or compositional fluctuations (*i.e.*, unstable domains); (iii) nanoscopic domains; and (iv) macroscopic domains. Domain stability and size depends on the line tension *γ*, which defines the free energy of the domain boundary (see *e.g.* ref. 15). That is, critical domain fluctuations occur at *γ* = 0. At small *γ*, nanoscopic domains are formed, whereas at large *γ* domains may grow to several microns in size.

Cholesterol is highly abundant in mammalian plasma membranes, and is a very peculiar membrane lipid. Although weakly amphiphilic, it has a finite solubility in phospholipid membranes, beyond which it precipitates from the bilayer as cholesterol monohydrate crystals.^16^ In bilayers composed of saturated or monounsaturated chains, cholesterol's solubility limit depends strongly on the phospholipid headgroup, and can be understood in terms of the “umbrella model”, where headgroups of neighboring lipids reorient to cover cholesterol's nonpolar surface, preventing its unfavorable exposure to water.^17^ The ability of different phospholipids to shield cholesterol should therefore depend not only on headgroup size, but also on chain packing considerations. Indeed, a 3- to 4-fold reduction in cholesterol solubility has been found in highly unsaturated PC bilayers composed of arachidonoyl (C20:4) or docosahexaenoyl (C22:6) chains at both the *sn*-1 and *sn*-2 positions,^18^ and several studies have shown that cholesterol preferentially interacts with membrane lipids composed of disaturated acyl chains.^19^

In binary lipid mixtures, cholesterol is well-known for its ordering effect on the fluid lamellar phase (L_α_), leading to the liquid-disordered (L_d_) and liquid-ordered (L_o_) phases at low and high cholesterol contents, respectively. On the other hand, lamellar gel phases (L_β_) are disordered by cholesterol.^10,20^ (Note, that frequently L_d_ is used synonymously with L_α_.) In describing the differences between these phases it is instructive to consider the two types of order that define the lamellar phases, namely translational or in-plane positional order (the spatial correlation between one lipid and another), and the chain configurational order of an individual lipid. These types of order are related to observables like the diffusion coefficient (translational order), hydrocarbon chain thickness and *gauche*/*trans* isomerization ratio (chain configurational order), all of which are strongly coupled in the L_α_ and L_β_ phases. In other words, low translational order is accompanied by low configurational order within fluid phase bilayers, and *vice versa* in the case of gel phase bilayers. Cholesterol, however, has the unique property of decoupling these two types of order: the L_o_ phase has very high chain order, but lacks long-range positional order. Properties of the lamellar phases are summarized in Fig. 2.

**Fig. 1:**
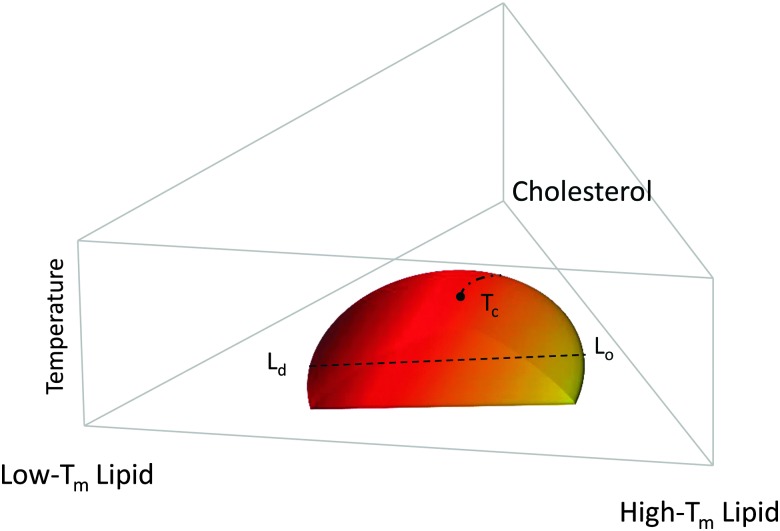
Generic compositional phase diagram for a ternary lipid mixture focusing on the temperature behavior of the L_o_/L_d_ coexistence regime. The dashed line indicates a tie-line, and the dashed-dotted line describes the critical transitions occurring at *T*_c_. *T*_m_ is the melting temperature. Other phase coexistence regions are not shown for purposes of clarity.

Fig. 1 shows a typical compositional phase diagram for raft-like ternary lipid mixtures of low-melting lipids (mainly di- or monounsaturated lipids), high-melting lipids (long chain disaturated phosphatidylcholines or sphingomyelin) and cholesterol. In raft-like lipid mixtures, as shown in Fig. 1, L_o_ and L_d_ phases coexist over an extended range of compositions and temperatures. Since L_o_ and L_d_ are fluid phases, their *γ* is isotropic, leading to the formation of circular domains. Demixing occurs along tielines, and the L_o_/L_d_ composition can be read off the tieline endpoints where they cross the phase coexistence boundary. The fraction of L_o_ or L_d_ changes along the tieline, and can be determined using the lever rule.^6^ The direction of tielines may differ from system to system, but in general shows that L_d_ domains contain most of the low-melting lipid, whereas L_o_ domains are enriched in the high-melting lipid, and moderately enriched (2- to 3-fold) in cholesterol.

**Fig. 2:**
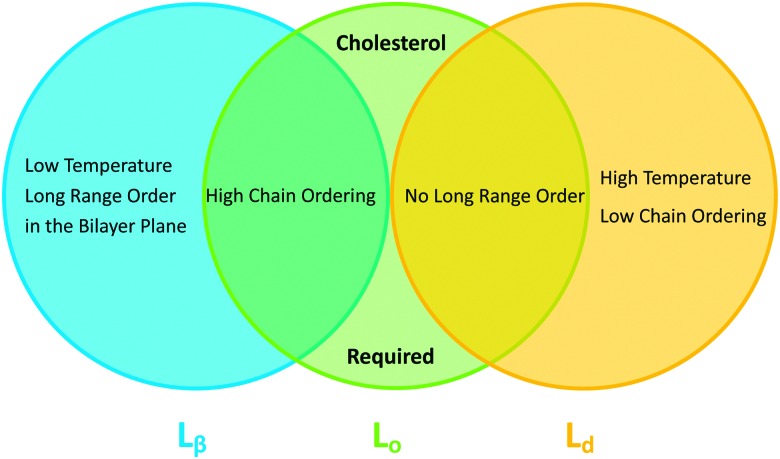
Venn diagram of properties shared between the gel (L_β_), liquid disordered (L_d_) and liquid ordered (L_o_) phases.

At high temperatures, L_o_ melts into a pure L_d_ phase, giving the phase coexistence regime a dome-like structure. If this melting occurs at the peak of the “dome” it passes through a critical point *T*_c_. Similarly, upon increasing cholesterol concentration, the L_d_ phase melts into an L_o_ phase. In this case, the tielines collapse into a single point, and the transition becomes second order. Thus, different critical transitions can be realized in ternary lipid mixtures, as shown in Fig. 1.

In the following section we describe how X-rays and neutrons can be used to probe overall domain size, as well as internal static and dynamic structures. For example, such information is needed for understanding how domains couple to protein partitioning and function. It is important to note that no bulky labels, which can potentially influence phase behavior,^21–23^ are needed for the scattering studies described herein.

## 3 General scattering theory

Even though X-rays are electromagnetic waves and neutrons particle waves, a single scattering theory is used to address both types of experiments. However, there are some important differences that must first be considered. To begin, X-rays interact with electrons, while neutrons interact with nuclei. Although not immediately obvious, X-ray scattering varies predictably with atomic number – heavy atoms scatter more strongly than lighter ones – while neutron scattering power varies erratically with atomic number. Importantly, however, neutrons are differentially sensitive to an element and its isotope(s). For example, hydrogen, which is ubiquitous in biological samples, has a coherent neutron scattering length *b*cohH = –3.7423 fm, while its stable isotope, deuterium, has *b*cohD = 6.674 fm. This difference between the two nuclei forms the basis of neutron contrast variation studies of biological materials. Therefore, by changing either the external contrast (by varying the H_2_O/D_2_O composition of the aqueous buffer), or by selectively deuterating specific parts of the biomolecule of interest,^24^ one can highlight or suppress static and dynamic structural features.

Another important difference between X-ray and neutron scattering relates to instrumental resolution. The wavelength spread Δ*λ*/*λ* at third generation synchrotron small-angle X-ray scattering (SAXS) beamlines is of the order of 0.01%, approximately 2 orders of magnitude finer than what is encountered at neutron beamlines. The main reason for this difference is the relatively low flux of neutron instruments, compared to X-rays, requiring monochromators capable of accepting a broader range of neutron wavelengths (*i.e.*, less monochromatic beams). An obvious consequence of this, is that SAXS peaks are significantly sharper than peaks from small-angle neutron scattering (SANS) instruments. This offers the possibility to perform line-shape analysis using SAXS, resulting in the bilayer's elastic constant (see below). A less obvious result of tighter collimation and increased monochromicity relates to the beam coherence volume *V*_coh_, which is described in terms of partial coherence in the theory for optics.^25^ *V*_coh_ has a longitudinal component, *i.e.* parallel to the propagating wave train,2
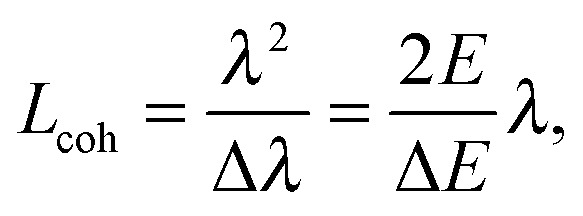
where Δ*E*/*E* is the energy resolution of either the neutron or X-ray beam, and two transverse components *T* *i*coh which vary inversely with the source aperture size.^26,27^ Typical values for *L*X-raycoh at synchrotron beamlines are on the order of 1 μm, while *L*neutroncoh ≤ 0.05 μm. The coherence volume is an important consideration for both transverse and in-plane bilayer structure determination, as will be discussed later on.

There is a third important difference between neutrons and X-rays. Neutron energies are typically on the order of meV, which are well within the range of thermally excited molecular motions, while X-rays are usually on the order of keV. Thus, while coherent inelastic X-ray scattering experiments on lipid membranes are feasible,^28,29^ neutrons are better suited for this purpose.^30^

### 3.1 Elastic X-ray and neutron scattering

In the case of elastic scattering there is no transfer of energy. It is therefore sufficient to consider the change in scattered intensity as a function of the momentum transfer vector, **q**. The magnitude of the scattering vector is given by *q* = 4π sin(*θ*)/*λ*, where *λ* is the X-ray or neutron wavelength, and 2*θ* is the angle between the incoming and scattered beams (*i.e.*, the scattering angle). Coherent elastic scattering of neutrons or X-rays provides information regarding spatial correlations of nuclei or electrons, respectively. However, unlike a crystal, where atoms are restricted to small thermal vibrations around well-defined positions, the inherent disorder of fluid lipid membranes prevents structure determination at atomic resolution. Thus it has proven useful to sum up the electrons or neutron scattering lengths per unit volume, and introduce the concept of the electron density profile (EDP) or neutron scattering length density (NSLD) profile (see Section 5).

Spatial correlations are contained in the amplitudes of the scattered wave or form factor *F*(**q**). *F*(**q**) is the sum of the coherent scattering length (*b* ^coh^) of all atoms in the sample (eqn (3)), and is proportional to the observed intensity of the scattered wave (eqn (4)):3
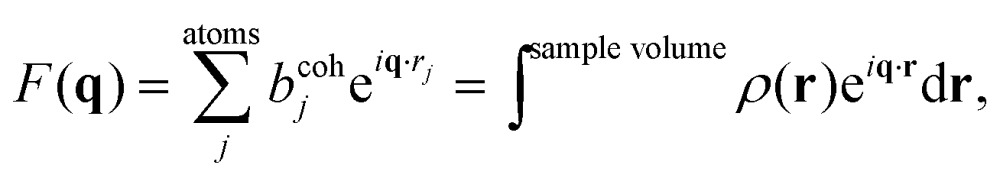

4 *I*(**q**) ∝ |*F*(**q**)|^2^.

The real-space distribution of the scattering lengths (the scattering length density, *ρ*) is the Fourier transform of the form factor,5
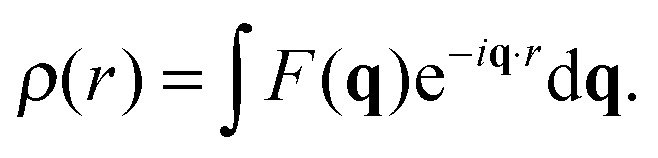
Membrane structural parameters can be determined from *ρ*, as discussed in Section 5.

Two types of positional correlations can occur, which are accounted for by a modification of eqn (4):

6 *I*(**q**) ∝ |*F*(**q**)|^2^*S*(**q**).

In concentrated solutions, unilamellar vesicles can interact through Coulomb or steric forces. This gives rise to an inter-particle (liquid) structure factor (*S* = *S*_p_(**q**)), which describes the relative positions of particles, and can be formulated by a variety of theories.^31^ In multibilayer stacks, membranes are positionally correlated along the bilayer normal, as in a 1D crystal. In this case, scattering is treated in terms of a lattice and a base, similarly to diffraction. The lattice is described by an intra-particle (crystal) structure factor (*S* = *S*_*i*_(**q**)) accounting for interactions between the sheets that give rise to long-range order (and hence Bragg peaks), while the base at each lattice point is given by eqn (3). In the case of fluid L_α_ phase lipid multibilayers, true long-range order breaks down due to pronounced bilayer bending fluctuations. This results in quasi long-range order, where positional correlations are described by a power law,^32^ leading to the characteristic cusp-like peak shape that is described by Caillé theory.^33,34^ For multilamellar vesicles (MLVs), the structure factor is given by^35^
7
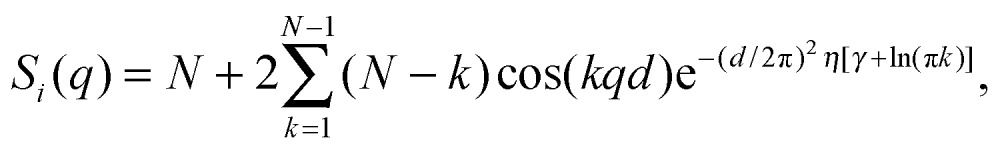
where *N* is the number of layers per scattering domain, *d* the lamellar repeat distance, and *γ* is Euler's constant. (We note that the magnitude of the scattering vector **q** can be used due to orientational averaging in MLVs.) Of particular importance is the Caillé or fluctuation parameter8
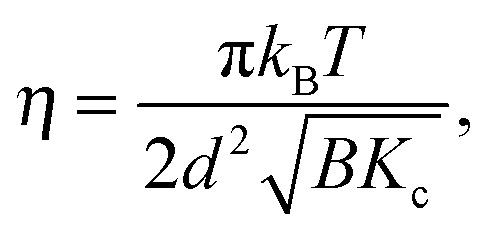
which is a function of the bulk modulus of compression *B* and the bilayer bending modulus *K*_c_^34^ (*k*_B_ is Boltzmann's constant and *T* temperature).

*S*_p_(**q**) and *S*_*i*_(**q**) are conceptually very different structure factors. Since nearest neighbors are typically much farther apart than bilayers in MLVs, contributions from *S*_p_(**q**) will occur only at very low scattering angles and may even be neglected in data analysis by exempting the low-angle regime. Contributions from *S*_*i*_(**q**) in turn occur at higher *q*-values and cannot be omitted when analyzing MLV data. Certainly, inter-particle correlations also occur in concentrated MLV solutions. However, due to the strong scattering power of *S*_*i*_(**q**) as compared to *S*_p_(**q**), such contributions are typically not observed.

### 3.2 Inelastic scattering

In contrast to the elastic scattering experiments described above, inelastic scattering results in the transfer of energy and momentum between the incident particle and the sample. Inelastic scattering of neutrons is ideal for studies of molecular motion in lipid bilayers, though its potential is relatively unexploited to date. The incident energy of neutrons typically used in inelastic scattering experiments is on the order of meV, comparable to the time scale of many processes in soft matter systems. For lipid bilayers, these include diffusion, vibration, molecular reorientation (*e.g.*, methyl rotation), lipid rotation, bilayer undulation, and bilayer thickness fluctuation. Inelastic X-ray scattering experiments are also feasible in lipid bilayer systems, but their use has been restricted to the study of collective vibrational dynamics^28,29^ due to the coherent nature of X-ray scattering and their relatively high incident energies (0.1–100 keV). An alternative, indirect route to study membrane dynamics has been recently achieved using time-resolved elastic X-ray diffraction on multibilayers coupled to a surface acoustic wave generator.^36^ Thus, by taking advantage of the ps-time structure of highly brilliant photon pulses at synchrotron facilities, the response of membranes to external oscillatory excitation can be exploited.

The goal of inelastic scattering experiments is to measure two quantities, namely the momentum transfer, **q** = **k**_f_ – **k**_*i*_, and the energy transfer, ℏ*ω* = *E*_f_ – *E*_*i*_. Here, **k**_*i*_ and **k**_f_ are the incident and scattered wave vectors, respectively, and *E*_*i*_ and *E*_f_ are the incident and scattered neutron energies, respectively. Through these two quantities, one can extract detailed information with respect to the frequency and geometry of atomic motions within a lipid bilayer.

The earliest inelastic scattering experiments were performed in the 1950s by Bertram Brockhouse^37^ at the then Chalk River Nuclear Laboratories using his newly developed triple-axis spectrometer. This novel way of measuring inelastic scattering enabled the measurement of scattered intensity at specific points in *q* and *ω*. A range of specialized spectrometers have subsequently been designed to optimize observation of scattered intensity simultaneously at multiple points in phase space, including time-of-flight,^38^ backscattering^39^ and neutron-spin-echo (NSE) spectrometers.^40^ This modern suite of instruments is able to probe motions on timescales ranging from 10^–14^ s to 10^–7^ s, and over length scales from 10^–7^ m to less than 10^–10^ m.

A quantitative description of inelastic scattering^41–43^ requires us to consider the basic quantity measured by neutron scattering experiments, namely the double differential cross-section:9
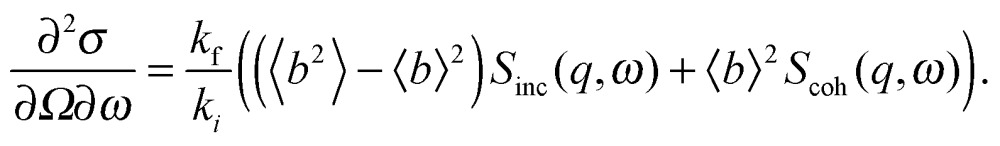
When multiplied by the number of incident neutrons, this quantity yields the number of neutrons scattered into a solid angle element ∂*Ω* with an energy transfer ℏ*ω*. The scattering length of the sample is given by *b*, and *S*(*q*,*ω*) is the dynamic structure factor. This relation brings to the fore the other major difference between neutron and X-ray scattering, namely the presence of both incoherent and coherent scattering. The separate dynamic structure factors, *S*_coh_(*q*,*ω*) and *S*_inc_(*q*,*ω*), describe these two classes of scattering. Each is connected to the microscopic motions of atoms in the sample, but in different ways. Coherent inelastic scattering probes the collective dynamics of an ensemble of atoms and is related to the double Fourier transform in space and time of the density–density correlation function:10
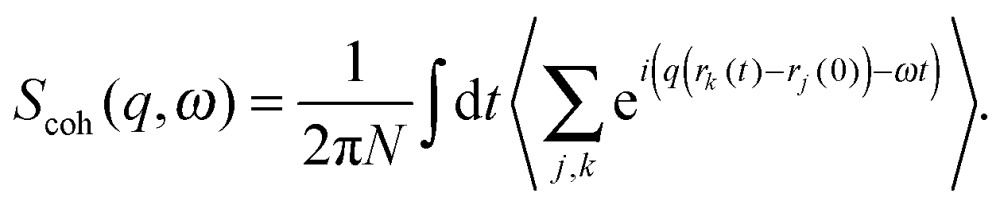

*S*_coh_(*q*,*ω*) therefore represents the probability of finding pairs of atoms at the same relative distance from each other after time *t*. The incoherent scattering function, *S*_inc_(*q*,*ω*), probes the motion of individual atoms and thus reflects the probability of finding an atom at a time *t*, within a distance *r* from of its initial position. *S*_inc_(*q*,*ω*) is given by the double Fourier transform in space and time of the self-correlation function:11
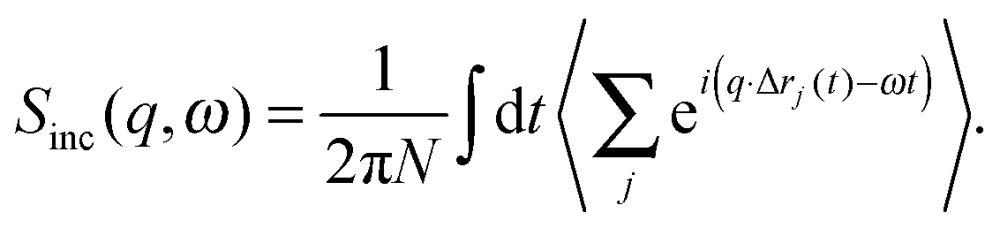
Eqn (11) relates the scattering to motions of individual atoms, and therefore has a more straightforward interpretation than *S*_coh_(*q*,*ω*). This is especially clear for the case of harmonic motions within a single potential well. This is a special case where a mean square displacement can be directly extracted^44^ from the elastic intensity for a given instrumental resolution.

The most common type of inelastic scattering measurement for biological materials focuses on the incoherent scattering from hydrogen. Hydrogen has an incoherent scattering cross-section of 80.27 barns, 40 times greater than that of deuterium, and more than 100 times larger than the other elements typically found in lipid bilayers: C (0.001 barns); N (0.5 barns); O (0.0008 barns); and P (0.005 barns). Because of the large incoherent scattering from hydrogen, incoherent scattering experiments often use protiated or partially deuterated lipids, hydrated with D_2_O in order to isolate the scattered signal from the lipid component of interest within the sample.^45–52^ Naturally, this situation can be reversed to study the dynamics of hydration water using a deuterated bilayer.^51,53,54^

The scattered intensity is customarily reduced to a function of *ω* for a set of *q* values, analysis of which yields information about the confinement geometry and relaxation times of atomic motions within the sample.^55^ The geometric information for a given dynamic process is usually extracted from the ratio of elastic intensity to total scattered intensity, and is represented as a phenomenological quantity called the Elastic Incoherent Structure Factor or EISF(*q*,*ω*). Numerous functional forms of the EISF have been put forward in order to accurately model the various atomic motions probed by scattering experiments.^56^

The inelastic scattering associated with a given dynamic process, *i*, is often modeled with a Lorentzian function *Γ*(*q*,*ω*), which is scaled by a factor *P*_*i*_ representing the fraction of hydrogen atoms participating in the *i*th process. This inelastic contribution of each process is combined with the EISF and a delta function *δ*(*ω*) to account for elastic scattering, to generate a theoretical scattering function including *n* processes:12
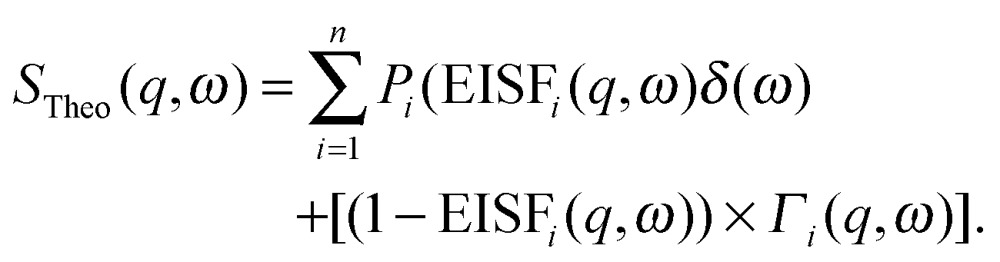
This function can then be fit against experimental data:

13 *S*_Exp_(*q*,*ω*) = DWF(*q*) × [*S*_Theo_(*q*,*ω*) ⊗ *R*(*q*,*ω*) + *B*(*q*,*ω*)],

where ⊗*R*(*q*,*ω*) indicates a convolution with the instrumental resolution function, *B*(*q*,*ω*) is an instrument background term, and DWF(*q*) is the Debye–Waller factor.

Deuterated molecules are also useful to study inelastic coherent scattering by reducing the overwhelming incoherent signal from hydrogen. This class of experiment excels in studies of lattice dynamics,^57,58^ but can also be useful in the study of collective motions of soft matter.^59–63^ Treatment of coherent scattering data is somewhat more complicated due to its sensitivity to pair-correlations. On the other hand, this sensitivity is responsible for the key feature of inelastic coherent scattering measurements, namely the ability to observe which atomic spacings are preserved during a particular collective motion. Borrowing from the polymer^64,65^ and protein^61^ literature, this information can be accessed by plotting the scattered intensity as a function of *q*, at a set of *ω* values, and comparing to the static structure factor, *S*(*q*,0). When a set of atoms moves collectively, maintaining their relative spacing, they will give rise to excess intensity at the associated *q* value, which can be expressed as:

14 *S*(*q*,*ω*) = *A*(*ω*) × *S*(*q*,0) × *q*^2^ + *B*(*ω*) × *q*^2^ + *C*.

Here, the first term represents the excess scattering from pair correlations that are preserved during a motion at a given *ω*, the second term represents the *q* ^2^ dependence of incoherent and out-of-phase motions, and the third term accounts for any *q*-independent multiple scattering. This relationship does not hold for atomic spacings in *S*(*q*,0) which are violated during a particular motion, indicating which atom pairs are moving together and which are not.

Analysis of neutron spin echo (NSE) data requires a different approach. The primary distinction of NSE, compared to the other inelastic techniques, is that it measures the intermediate scattering function, the ISF or *I*(*q*,*t*), rather than the dynamic structure factor, *S*(*q*,*ω*). The ISF is typically reported as *I*(*q*,*t*)/*I*(*q*,0) so that the quantity is normalized to 1. *I*(*q*,*t*) is simply the Fourier transform of the dynamic structure factor in the time domain. Another difference is that analysis of NSE results is typically performed in the time domain, using peak functions (rather than decay functions) to fit data.

Although NSE is capable of probing slow diffusive motions of lipids and bilayer thickness fluctuations, the most common spin echo experiments on lipid bilayers are direct measurements of bilayer undulation, allowing access to the bilayer's bending modulus.^66–71^ Typically, coherent scattering in the range 0.05 < *q* < 0.2 Å^–1^ is analyzed using a modification^66,70,72^ of the approach put forward by Zilman and Granek.^73^ Briefly, the ISF is fit in the time domain using a stretched exponential decay:15
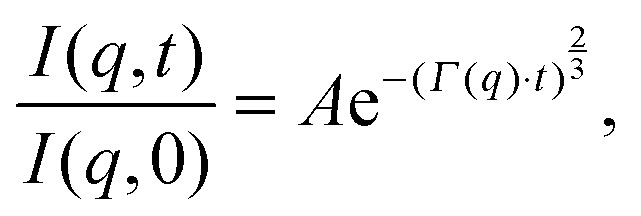
where *A* is a normalization constant (typically set to 1) and *Γ*(*q*) is the relaxation rate, related to the bilayer bending modulus *K*_c_ through:16
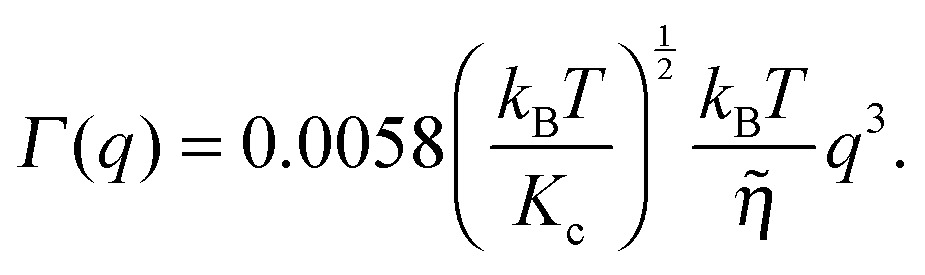
(*N.B.*: here, *η̃* is the solvent viscosity, not to be confused with the Caillé parameter in eqn (8)). Eqn (16) implies that a plot of *Γ*(*q*)/*q* ^3^ as a function of *q* will exhibit a constant value that is inversely proportional to the square root of *K*_c_.

## 4 Sample geometries

As discussed, lipid domains can be studied using a variety of scattering techniques, some of which demand unique sample preparations, conditions and geometries. From the standpoint of biological relevance, unilamellar vesicles (ULVs) are the most desirable mimics of a cellular membrane. Diffuse scattering from a dilute ULV suspension affords the possibility to extract the bilayer's continuous *F*(**q**) (eqn (4)), and often offers extended ranges for the scattering vector's transverse component (**q**_*z*_).

Arguably the easiest method of sample preparation is that of MLVs, whereby a dry lipid mixture film is hydrated with water. Measurement of MLVs results in the presence of a *F*(**q**) and a *S*_*i*_(**q**) as a convolution of both the radial and in-plane heterogeneities of the bilayer structure. A great deal of information can be extracted from MLV samples, including (but not limited to) the bilayer's stiffness, and the presence of domains (Section 6).

**Fig. 3:**
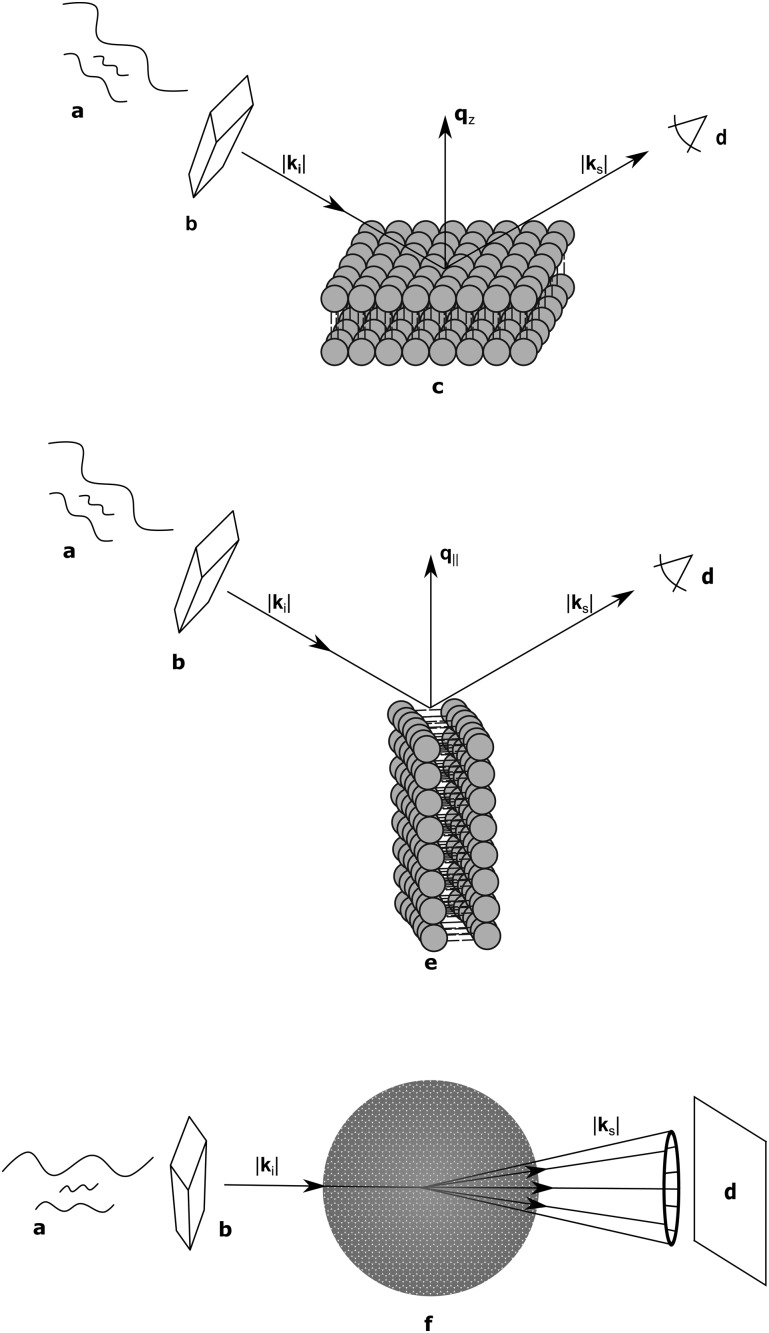
Schematic diagram of bilayer scattering geometries. *Upper*, a monochromatic beam with wave vector is selected from a “white” beam of incident neutron or X-ray radiation (a) using a monochromator (b). The angle of the scattered wave vector (where for elastic scattering) is recorded by a detector (d). The sample (c) is oriented such that the scattering vector is perpendicular to the bilayer surface, and therefore probes transverse bilayer structure. *Middle*, a 90° sample rotation (e) results in a scattering vector that is parallel to the bilayer surface, allowing for interrogation of in-plane structure. *Bottom*, a vesicle sample (f) results in isotropic scattering, whereby and are probed simultaneously.

Supported samples can be prepared as a single bilayer (typically examined with reflectometry) or as multilamellar stacks for interrogation by diffraction techniques. Although MLVs are themselves aligned bilayers, alignment on a solid substrate allows for the transverse and lateral structures to be examined independently (Fig. 3). The separation of **q**_*z*_ and **q**_∥_ (the lateral scattering vector component) allows for the unambiguous assignment of scattering features arising from the different orientations. Like all systems, solid-supported bilayers suffer from some drawbacks. For example, supported lipid bilayers have proven difficult to fully hydrate,^74,75^ though recent advances in sample environments have achieved hydration levels of better than 99.6% as determined by lamellar repeat spacings.^76^ Perturbations attributed to bilayer–substrate interactions are limited to the first few bilayers, although much effort has been expended into functionalizing the substrate surface with a polymer cushion for use in single bilayer studies.^77^

The aforementioned sample conditions are characterized by low resolution data, however, improved structural data can be achieved by utilizing the neutron scattering method of contrast variation. The ability to change contrast conditions without resorting to bulky and unnatural probes that can alter the bilayer's physical properties is one clear advantage elastic neutron scattering has over other biophysical techniques, including X-ray scattering.^78^ Manipulating contrast is particularly important since the scattering intensity is proportional to the square of the SLD difference between the sample and solvent (medium). Contrast can be systematically changed by substituting one isotope of an element with another (discussed above). In the case of biological samples, the substitution of hydrogen for deuterium is commonly used to vary contrast. Scattering from individual components of the system, such as phase separated regions of a vesicle, can be suppressed through contrast matching with the solvent, allowing for the determination of lateral structure and composition. Contrast variation in a SANS experiment on lipid domains is illustrated below (Section 6.1) (Fig. 7).

## 5 Homogeneously mixed bilayers: a brief update

Although homogeneously mixed fluid bilayers lack long range in-plane atomic correlations, they do possess one-dimensional out-of-plane correlations. The structure of a homogeneous fluid bilayer can therefore be thought of as the time-averaged distribution of matter projected onto the bilayer normal. A scattering experiment provides a distorted reflection of this matter distribution, where features are reshaped by the relative interaction strength of the probe (neutrons or X-rays) with the lipid's chemical makeup. In this sense, the real-space scattering length density profiles obtained from different types of scattering experiments (*i.e.*, X-ray data, or different contrast neutron data) are simply different representations of the bilayer's structure averaged over time/energy. While traditional bilayer structural analyses model SLD profiles of standalone scattering data,^35,79–83^ a model based on matter density distribution can easily combine different contrast data sets (*i.e.*, X-ray and neutron) into a single global analysis, resulting in a more robust bilayer structure.

White and coworkers were the first to exploit this fundamental link between the bilayer's different structural representations, in their development of the “composition space model”. Because individual atoms are not well-localized in a thermally disordered bilayer, they are best described by broad statistical averages. King and White^84^ proposed a coarse-grained lipid structure, where neighboring atoms are grouped into quasi-molecular distributions whose atomic number density profiles are described by simple functional forms (*e.g.*, uniform or Gaussian distributions). A fully resolved fluid bilayer structure consists of a handful of such quasi-molecular distributions, typically 2–3 to describe the lipid headgroup, and 3–4 to describe the hydrocarbon chain region. Scattering length density profiles for different contrast data sets are then obtained by scaling the component number density distributions with an appropriate scattering length (*i.e.*, the sum of individual atomic scattering lengths making up the distribution). Through the joint refinement of neutron and X-ray diffraction data, Wiener and White determined the fully resolved structure of a partially dehydrated fluid DOPC bilayer.^85–89^

**Fig. 4:**
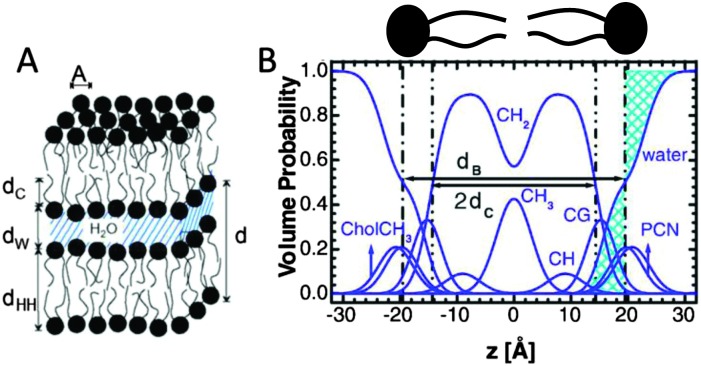
Description of membrane structure in terms of the SDP model. Panel A shows a schematic of a stack of membranes with the corresponding structural parameters: *d* – lamellar repeat distance; *d*_B_ – bilayer thickness; *d*_W_ – bilayer separation; *d*_C_ – hydrocarbon chain length; *d*_HH_ – headgroup-to-headgroup distance; and *A* – area per lipid. Panel B shows the volume distribution functions of quasimolecular distributions in terms of the SDP model. Figure adapted from ref. 91.

Kučerka *et al.* extended this approach with their Scattering Density Profile (SDP) analysis (Fig. 4), which leverages the atomistic detail of MD simulations to guide the choice of atomic groupings, thereby maximizing the model's compatibility with different contrast X-ray and neutron data.^90^ For this model, which uses data from ULVs, eqn (3) becomes17
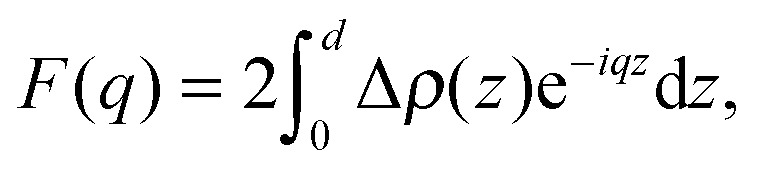
with18
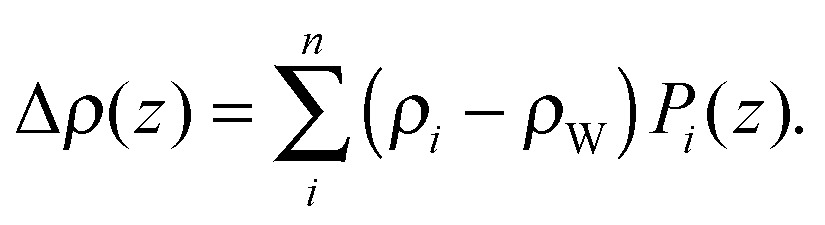



Here *P*_*i*_(*z*) represent the volume distributions of given molecular fragments, each described by a Gaussian or error function. A typical parsing scheme for a phosphatidylcholine bilayer would be, for example, the choline methyl (CholCH_3_), phosphate + CH_2_CH_2_N (PCN), carbonyl + glycerol (CG), hydrocarbon methylene (CH_2_) and terminal acyl chain methyl (CH_3_) groups. The *P*_*i*_'s are scaled by the contrast of their given scattering length densities, *ρ*_*i*_, with water, *ρ*_W_.

By combining SANS data at several D_2_O/H_2_O ratios (“external” contrasts) with SAXS data, Kučerka and coworkers obtained the first fully resolved bilayer structure from a vesicle suspension at full hydration. The SDP approach has since been used to determine structures for a wide range of biologically relevant lipids using fully hydrated fluid bilayers, including phosphatidylcholine,^91,92^ phosphatidylglycerol,^93^ phosphatidylserine,^94^ phosphatidylethanolamine,^95^ and cardiolipin.^68^ A major achievement of the SDP model is the robust determination of bilayer thickness, defined as19
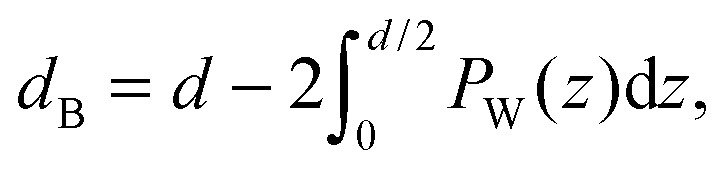
and area per lipid20
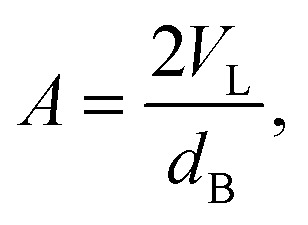
quantities that are crucial for the validation of MD force fields (reviewed in ref. 96). Here, *d* is the lamellar repeat distance, *P*_W_ is the volume distribution function of water, and *V*_L_ is the lipid's molecular volume, which can be independently obtained using a variety of techniques.^97^

**Fig. 5:**
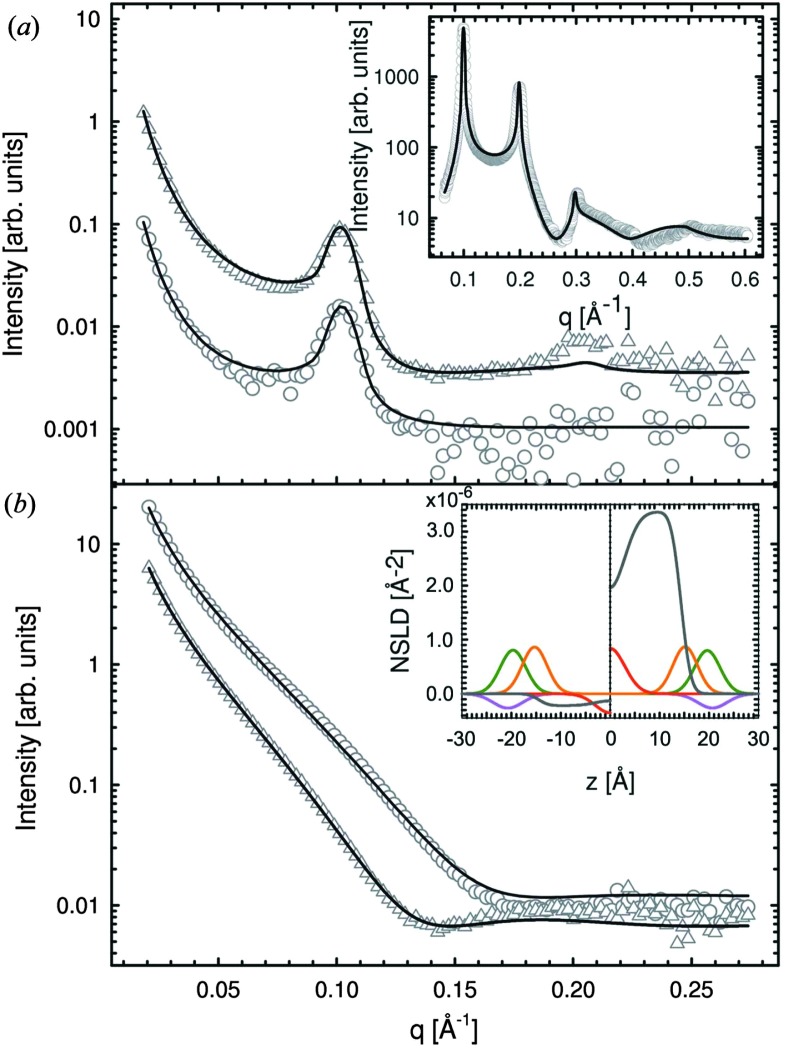
Joint analysis of SAXS (inset) and SANS data of POPC MLVs and ULVs. Panel A shows SANS data of POPC (circles) and chain deutrated POPC-d31 (triangles) MLVs. Panel B shows corresponding data for ULVs (same symbols). Figure is adapted from ref. 98.

Recently, Heftberger *et al.* ^98^ combined the SDP model with a Caillé structure factor (eqn (7)) to analyze MLVs in the L_α_ phase (Fig. 5). In this case, the scattered intensity is given by21
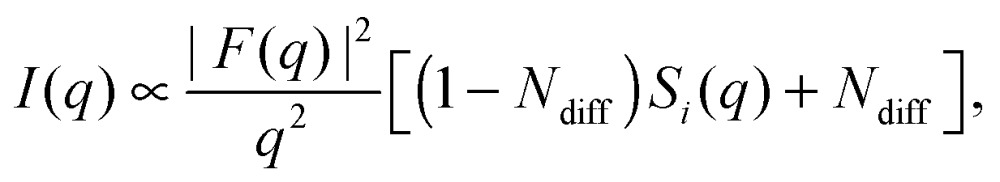
where *F*(*q*) is given by eqn (17) and *S*_*i*_(*q*) by eqn (7). The scalar *N*_diff_ accounts for the presence of positionally uncorrelated bilayers. An advantage of this hybrid model is that membrane structure can be studied at SDP resolution without the need for extruded ULVs. Further, by using the structure factor, an experimental window on membrane fluctuations (eqn (8)) becomes accessible, opening new opportunities to study bilayer interactions and membrane mechanical properties (see Section 6.3). Finally, recent attempts strive to further increase membrane structural resolution by using atomically detailed SLD models.^99^

## 6 Phase separated bilayers

**Fig. 6:**
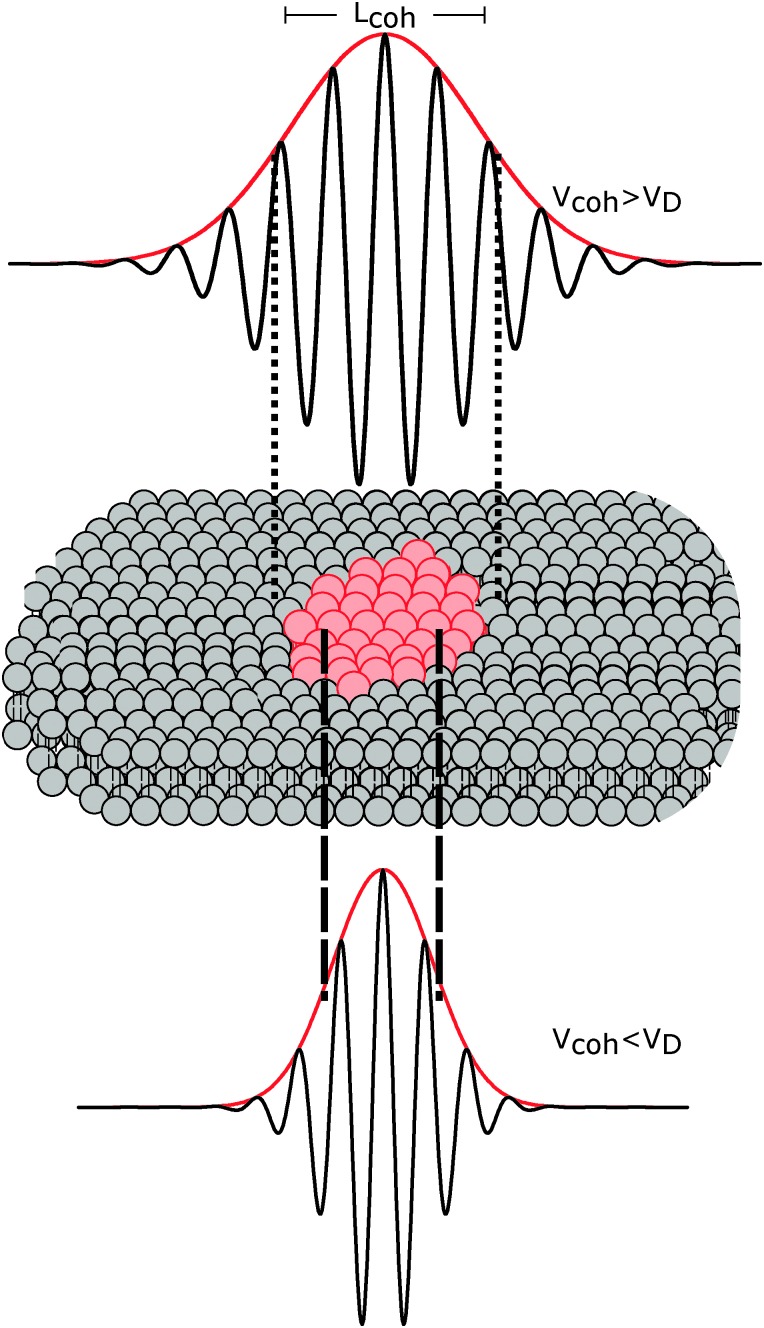
The influence of coherence volumes in detecting membrane domains. Coherence is represented as a 1D interferrogram with a given coherence length *L*_coh_ (see also eqn (2)). For low wavelength spread and large *V*_coh_ (top), scattering contributions from the domain and surrounding bilayer add coherently (eqn (22)). In this case, domain size, morphology, and configuration can in principle be determined in a small-angle scattering experiment. For multibilayer samples, Bragg peaks from distinct L_o_ and L_d_ lattices are averaged. At high wavelength spread (bottom), *V*_coh_ < *V*_D_, resulting in incoherent addition of domain scattering contributions (eqn (23)), and a superposition of L_d_ and L_o_ Bragg peaks in a SAXS experiment, as demonstrated in Section 6.3.

The importance of coherence volume *V*_coh_ was mentioned in Section 3. For phase separated systems in particular, the domain size—or more precisely, the domain volume *V*_D_—with respect to *V*_coh_ must be considered. If V_coh_ ≥ *V*_D_, domain scattering contributions add coherently (Fig. 6 top). For ULVs exhibiting two-phase coexistence, the observed scattering intensity is thus given by:

22 *I*(*q*) ∝ |*φ*_A_*F*_A_ + (1 – *φ*_A_)*F*_B_|^2^,

where *φ*_A_ is the fraction of phase A, and *F*_A_ and *F*_B_ are the form factors of phases A and B, respectively. If however *V*_coh_ < *V*_D_, the form factors add up incoherently (Fig. 6 bottom). Thus, for the same phase separated system we now have

23 *I*(*q*) ∝ *φ*_A_|*F*_A_|^2^ + (1 – *φ*_A_)|*F*_B_|^2^ + *Ĩ*(*q*),

where *Ĩ*(*q*) accounts for the coherent addition of form factors in the domain boundary regime, where both phases are present within a single *V*_coh_ element. The latter contribution (in-plane) is typically neglected in the analysis of transverse domain structure.^100^ Note also that both equations assume an infinitesimally sharp domain boundary, which if it were not the case would result in an additional contribution.

The effect of *V*_coh_ was demonstrated by Armstrong *et al.* ^101^ for dipalmitoyl phosphatidylcholine (DPPC) in the vicinity of its melting transition. Upon cooling from the liquid-disordered L_d_ phase, small gel-like domains begin to nucleate. Using neutron diffraction and oriented multibilayers, and by selectively detuning the pyrolytic graphite monochromator, the authors were able to decrease *L*_coh_ from 242 Å to 30 Å. Only for *L*_coh_ < 103 Å was phase coexistence observed.

With regard to domain size, another factor to consider is the overall ULV size. For 50–100 nm diameter ULVs, as studied by SANS (see Section 6.1), *V*_coh_ > *V*_D_ for L_o_/L_d_ phase coexistence, allowing for in-plane structure (*e.g.*, domain size and configuration) to be detected. In multibilayers, domains may grow to several microns. In such cases, *V*_coh_ < *V*_D_. This gives rise to two lamellar lattices from which one can measure each domain's transverse structure (see Section 6.3). However, things may differ for lipid mixtures exhibiting nanoscopic domains,^102^ where domain size is of the order of *V*_coh_.

### 6.1 Elastic neutron scattering – SANS

#### 6.1.1 Detecting domains

**Fig. 7:**
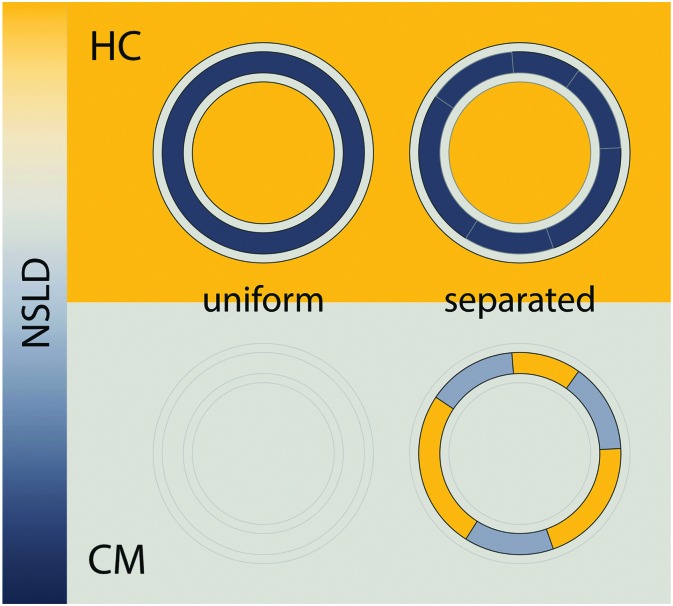
Detecting domains with neutron scattering requires optimizing contrast conditions. Neutron scattering length density (NSLD) is depicted as a continuous gradient between dark gray and yellow (left). The upper panel demonstrates a typical SANS experiment performed in 100% D_2_O solvent, using protiated lipids. In this “high contrast” (HC) scenario, a large NSLD difference exists between solvent and the lipid hydrocarbon region (with a smaller contrast between the lipid headgroup and hydrocarbon chains). As such, lateral segregation of lipids (*i.e.*, phase separation) results in no apparent change in contrast or scattered intensity (upper right). However, by using chain perdeuterated lipids and solvent contrast variation, it is often possible to simultaneously match the SLD of the lipid headgroup, hydrocarbon chains, and water, as shown in the lower panel. In such a “contrast matched” (CM) sample, uniform lipid mixing results in a null scattering condition (lower left), but lateral segregation of chain protiated and chain perdeuterated species generates significant lateral contrast (lower right), and hence an increase in scattering.

As discussed in Section 5, the combination of SAXS and SANS provides detailed information about the distribution of matter in the direction of the bilayer normal, allowing for the robust determination of lipid areas and thicknesses in homogeneous bilayers. Such studies rely on SLD differences between the solvent and bilayer – for SANS, a typical experiment uses fully protiated lipids in 100% D_2_O. Though optimal for studying transverse bilayer structure, these conditions largely mask the scattering signatures of lateral phase separation. As shown schematically in the upper panel of Fig. 7, a large solvent/bilayer contrast easily overwhelms any contrast generated by segregation of protiated lipids within the bilayer plane. Clearly, experimental conditions must be modified to suppress scattering arising from transverse contrast, and enhance scattering arising from lateral contrast.

Pencer *et al.* systematically addressed this problem by considering how the various SLD contrasts in a phase separated vesicle contribute to its total scattering signal.^81^ Approximating the vesicle structure as a series of concentric shells corresponding to the inner headgroups, hydrocarbon, and outer headgroups, the following SLDs are calculated:24
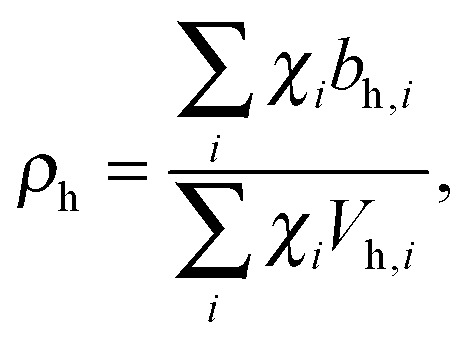

25
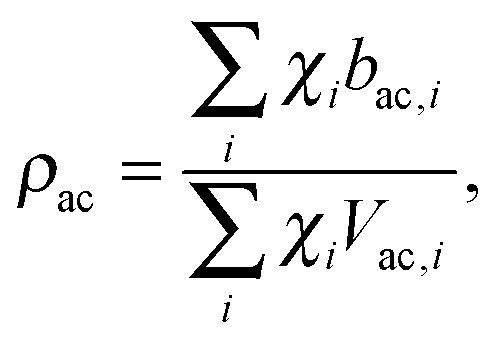
where the subscripts h and ac refer, respectively, to the headgroup and acyl chain shells, *b* is the coherent neutron scattering length, *V* is the molecular volume, and *χ*_*i*_ is the bilayer mole fraction of lipid species *i*. Similarly, the average total bilayer SLD is given by26
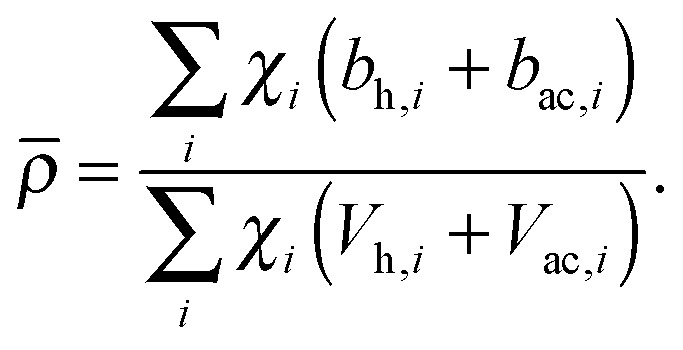



For ULVs, the total scattering 
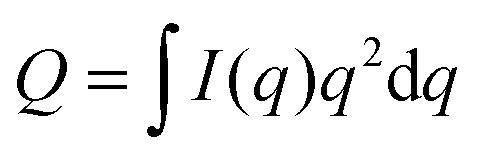
 (also called the Porod invariant) can be decomposed into three additive contributions related to: (1) the SLD contrast between the average vesicle composition and the solvent; (2) the radial SLD contrast between the lipid headgroups and acyl chains; and (3) the lateral SLD contrast arising from domains having a different average acyl chain composition. Defining these three respective contributions as *Q*_0_, *Q*_r_, and *Q*_l_ (*i.e.*, Q = *Q*_0_ + *Q*_r_ + *Q*_l_), Pencer *et al.* ^81^ showed that

27 *Q*_0_ ∝ (*ρ̄* – *ρ*_m_)^2^,

28 *Q*_r_ ∝ *t*_f_(1 – *t*_f_)(*ρ*_ac_ – *ρ*_h_)^2^,

29 *Q*_l_ ∝ *t*_f_*a*_f_(1 – *a*_f_)(*ρ*_L_d__ – *ρ*_L_o__)^2^,

where *ρ*_m_ is the solvent SLD, *ρ*_L_d__ and *ρ*_L_o__ are the respective acyl chain SLDs of the L_d_ and L_o_ phases, *t*_f_ = *t*_ac_/(*t*_ac_ + 2*t*_h_) is the ratio of the average acyl chain thickness to the total bilayer thickness, and *a*_f_ is the vesicle surface area fraction occupied by domains. Importantly, the total homogeneous scattering contribution *Q*_hom_ = *Q*_0_ + *Q*_r_ depends only on the solvent and averaged lipid SLDs, and not on the lateral distribution of lipids within the bilayer. In this sense, the homogeneous scattering is an undesirable background signal. The optimal experimental condition for detecting domains corresponds to enhancing *Q*_l_ and minimizing *Q*_hom_ through contrast matching.

An instructive example of such contrast matching is found in Heberle *et al.*,^102^ where the authors examined domain formation in a series of lipid mixtures including DSPC/DOPC/Chol in a 39/39/22 ratio. At 20 °C, this mixture separates into coexisting L_d_ and L_o_ phases, strongly enriched in DOPC and DSPC, respectively.^22^ Though DOPC and DSPC have similar acyl chain NSLDs (Table 1), a large contrast between L_d_ and L_o_ domains can nevertheless be generated by replacing DSPC with its chain perdeuterated counterpart, DSPC-d70. Because of its favorable partition into L_o_ domains, the use of DSPC-d70 results in a large increase in *ρ*_L_o__ but only a small increase in *ρ*_L_d__, thereby enhancing the lateral scattering contribution *Q*_l_ according to eqn (29). At the same time, the background homogeneous scattering *Q*_hom_ is also affected, through changes in the average acyl chain and bilayer SLDs (*ρ*_ac_ and *ρ̄*, respectively).

**Table 1:** Neutron scattering lengths, molecular volumes at 60 °C, and scattering length densities of various lipid species

Molecule	Chemical formula	*b* (fm)	*V* (Å^3^)	NSLD (fm Å^–3^)
PC headgroup	C_10_H_18_NO_8_P	60.1	331^*a*^	0.181
DSPC chains	C_34_H_70_	–35.8	1017^*b*^	–0.035
DSPC-d70 chains	C_34_D_70_	692.9	1017^*b*^	0.681
DOPC chains	C_34_H_66_	–20.8	1003^*c*^	–0.021
POPC chains	C_32_H_64_	–26.6	953^*b*^	–0.028
Cholesterol	C_27_H_46_O	13.3	630^*d*^	0.021
Water	H_2_O	–1.68	30.4	–0.055
Heavy water	D_2_O	19.15	30.5	0.628
34.6% heavy water	H_1.31_D_0.69_O	5.53	30.4	0.181

^*a*^ Ref. 104.

^*b*^ Ref. 91.

^*c*^ Ref. 105.

^*d*^ Ref. 106.

For the experiments described above, it is important to recognize that neither the domain nor the surrounding phase compositions are contrast matched to the solvent. Rather, it is the overall or average bilayer composition that is matched to solvent. This can be achieved by simple calculation provided lipid volumes are known (*e.g.*, Table 1 and eqn (24)–(26)), or experimentally by measuring a solvent contrast series to determine the total scattering minimum.^103^ Importantly, this contrast matching scheme does not depend in any way on *a priori* knowledge of domain composition: by design, a well-mixed bilayer with no mesoscale domain structure (*e.g.*, at temperatures above the upper miscibility transition) will exhibit minimal scattering. On the other hand, if the different lipid species segregate from each other into compositionally distinct domains, then neither phase is contrast matched to water, nor are they matched to each other. The resulting spatial contrasts (both lateral and transverse) result in increased scattering.

**Fig. 8:**
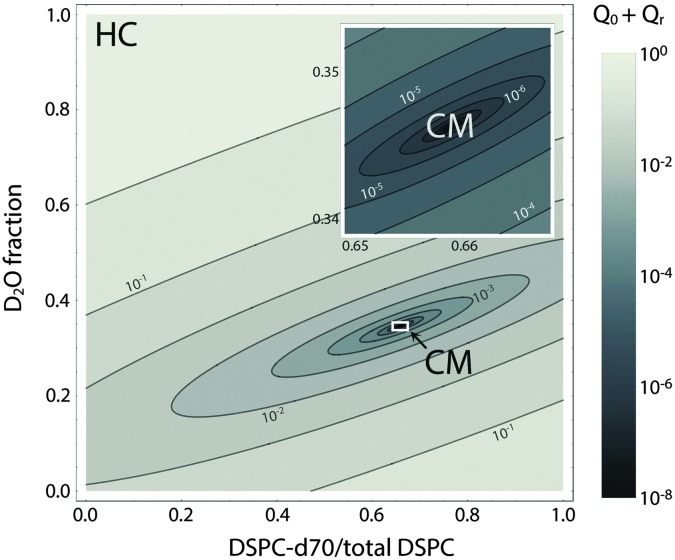
Optimizing experimental conditions for detecting domains in DSPC/DOPC/Chol. The relative homogeneous background scattering *Q*_hom_ = *Q*_0_ + *Q*_r_, calculated from lipid NSLDs (Table 1) using eqn (24)–(29), is plotted *vs.* fraction of DSPC-d70 (to total DSPC) and the solvent fraction of D_2_O. A global contrast match point is observed at 34.6% D_2_O and 65.9% DSPC-d70 (“CM”, expanded in inset). Close to the contrast match point, *Q*_hom_ is attenuated by >6 orders of magnitude relative to a fully protiated bilayer in 100% D_2_O solvent (“HC”).

Fig. 8 shows a contour plot of *Q*_hom_ *vs.* the fraction of DSPC-d70 (to total DSPC), and the solvent fraction of D_2_O calculated using eqn (24)–(29) and data from Table 1. A sharp minimum in *Q*_hom_ is observed at 34.6% D_2_O and 65.9% DSPC-d70, precisely the point where the solvent and average bilayer NSLDs are matched to the PC headgroup. Using these experimental conditions, *ρ*_m_ ≅ *ρ̄* ≅ *ρ*_h_ ≅ *ρ*_ac_ ≅ 0.181 fm Å^–3^: consequently, if the lipids are randomly mixed within the bilayer plane (*e.g.*, at high temperature), a null scattering condition exists (Fig. 7, lower left). However, demixing of saturated and unsaturated lipids (*i.e.*, DSPC and DOPC) causes lateral NSLD fluctuations that generate in-plane contrast (Fig. 7, lower right), resulting in increased scattering according to eqn (29).

**Fig. 9:**
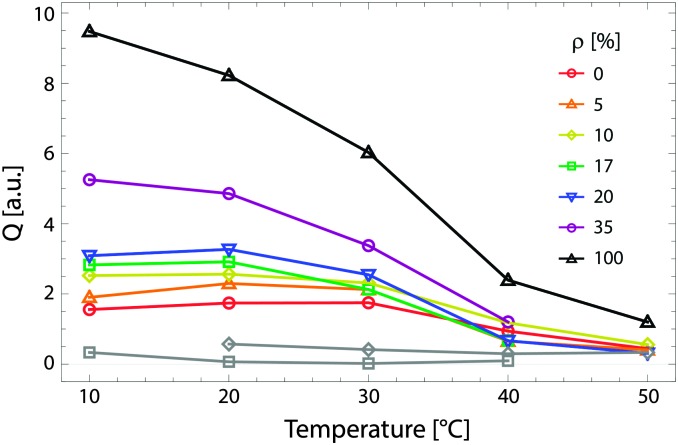
Experimentally measured total scattering reveals domain formation in 4-component lipid mixtures. Shown is the Porod invariant 
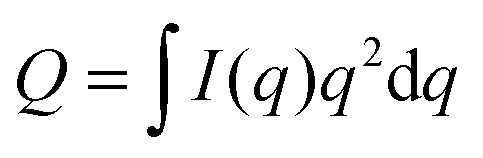
, plotted *vs.* temperature for DSPC/(DOPC + POPC)/Chol mixtures in a 0.39/0.39/0.22 molar ratio. Colors correspond to different values of the composition parameter *ρ* = *χ*_DOPC_/(*χ*_DOPC_ + *χ*_POPC_) as indicated in the legend. Also shown are two single-phase control samples: DSPC/POPC/Chol 0.325/0.325/0.35 (gray diamond) and POPC/Chol 0.65/0.35 (gray square). Figure adapted from ref. 102.

Fig. 9 shows the total scattering (*i.e.*, the Porod invariant *Q*) for several 4-component lipid mixtures studied at bilayer contrast matching conditions.^102^ For mixtures containing DSPC and low-melting lipid (either POPC or DOPC) in a 1 : 1 ratio, in addition to 22 mol% cholesterol, a marked increase in *Q* was observed with decreasing temperature, indicating domain formation. At fixed temperature, *Q* showed a systematic decrease as POPC replaced DOPC, consistent with a reduction in domain area fraction, and weaker DSPC partitioning between the L_d_ and L_o_ phases.^107,108^ In contrast, single phase mixtures showed low total scattering and little variation over the temperature range studied.

As a model-free method, the Porod invariant is a robust diagnostic tool for probing lateral bilayer inhomogeneities.^102,109^ However, this strength is at the same time a weakness – by collapsing the *q*-dependence of the scattering signal, any potential information regarding the size, shape, and spatial distribution of domains is lost. Elucidating these details requires modeling *I*(*q*), as will be discussed in the next section.

#### 6.1.2 Analytical form factor

An analytical solution for domain scattering was first provided by Anghel *et al.*,^110^ in which the authors used a spherical harmonic expansion of the scattering amplitude to derive the form factor of a vesicle containing a single round domain. However, this model proved inadequate for describing experimental SANS data in the well-studied domain forming mixtures DPPC/DOPC/Chol^111^ and DSPC/(DOPC + POPC)/Chol.^102^ In both studies, Monte Carlo analyses instead suggested the presence of multiple domains in ULVs. To facilitate the study of such systems, the analytical form factor was recently generalized to static configurations of multiple, arbitrarily sized domains, with the ability to accommodate distributions of domain sizes or configurations through appropriate averaging.^112^ To illustrate the model, we now consider the analytical solution for uniformly sized round domains.

The scattered intensity of a vesicle containing multiple domains can be expressed as:

30 *I*(*q*) = *I*_hom_(*q*) + *I*_intra_(*q*) + *I*_inter_(*q*).

The first term in eqn (30) comprises the homogeneous contribution to the total scattering, arising from radial SLD contrasts of each phase:31
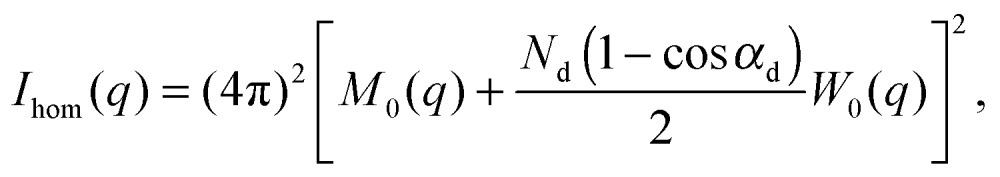

32
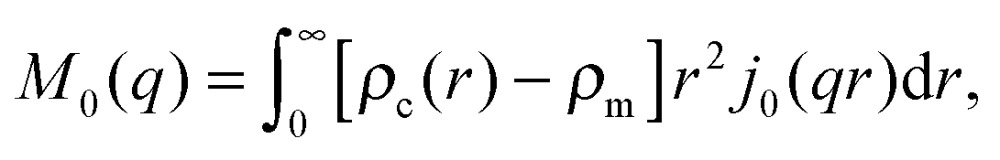

33
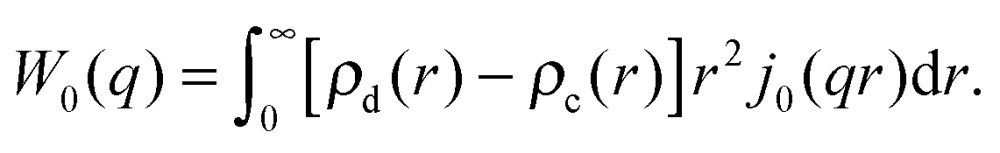
Here, subscripts d and c refer, respectively, to the domain and continuous phases, *N*_d_ is the number of domains, *α*_d_ is the angle formed by vectors pointing from the vesicle center to the domain center and edge, and *j*_0_ is the zeroth order Bessel function. Eqn (32) is recognized as the core/shell (*i.e.*, vesicle) form factor for the continuous phase, and is calculated as the Fourier transform of its radial SLD profile, while eqn (33) represents the Fourier transform of the radial SLD difference between the domain and continuous phases. The second term in eqn (30) describes intra-domain scattering arising from domain self-correlation:34
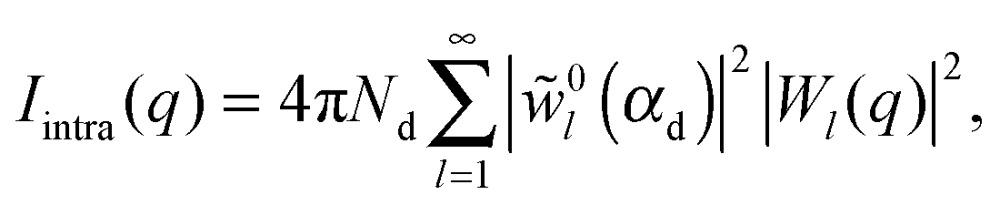

35
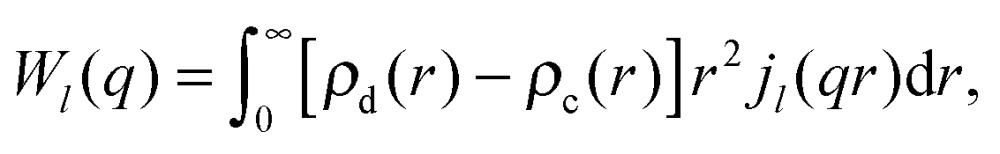

36
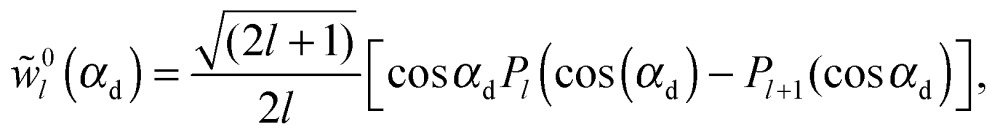
where *P*_*l*_ is the Legendre polynomial of degree *l*. Finally, the third term in eqn (30) accounts for inter-domain scattering, arising from coherent interference between different domains:37
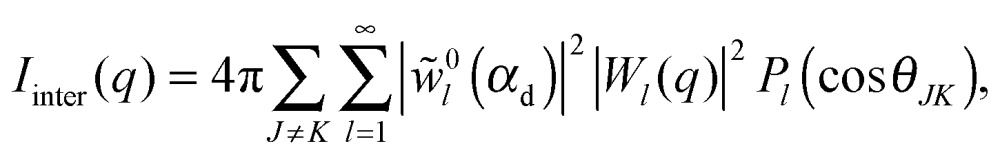
where *θ*_*JK*_ is the angle between the vesicle center and the centers of domains *J* and *K*. Eqn (37) reveals that the inter-domain scattering contribution depends solely on the relative spatial configuration of domain pairs.

**Fig. 10:**
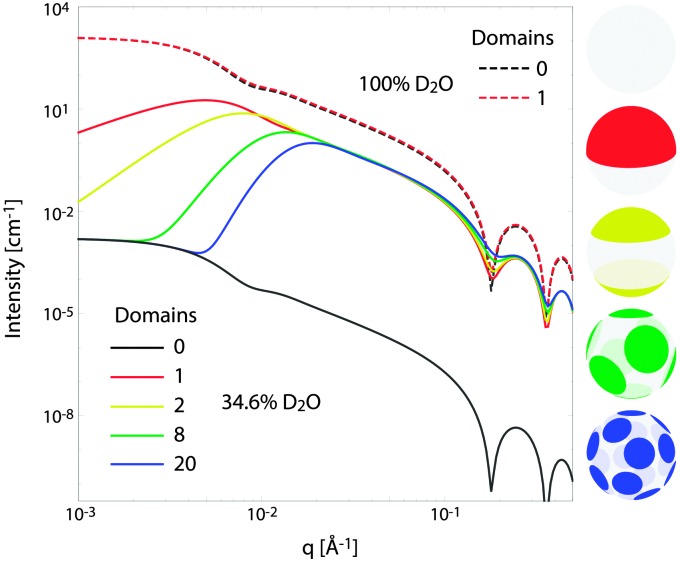
Theoretical scattering curves for multidomain vesicles. For all curves shown, the average bilayer NSLD is identical. Differences in scattered intensity are due to differences in solvent NSLD and/or the lateral NSLD distribution, as indicated by the figure legend and color-coded vesicle images (right), and described in the text.

Fig. 10 illustrates the analytical model for typical experimental conditions (*e.g.*, Table 1). For all theoretical curves, the average bilayer NSLD is identical (*ρ̄* = 0.18 fm Å^–3^), and differences in scattered intensity are due either to differences in solvent NSLD, or the presence (or absence) of domains. At 100% D_2_O (*ρ*_m_ = 0.636 fm Å^–3^, dashed curves), a large contrast exists between the solvent and bilayer; consequently, the homogeneous scattering dominates, and there is little apparent difference between uniform (black dashed) and phase-separated (red dashed) vesicles. However, consistent with the prediction of Fig. 8, the differences are greatly magnified near the contrast match point of 34.6% D_2_O (*ρ*_m_ = 0.181 fm Å^–3^, solid curves). While scattering from a uniformly mixed vesicle exhibits the same relative *q*-dependence at 100% and 34.6% D_2_O (black dashed and black solid curves, respectively), the total homogeneous intensity is attenuated by a factor of nearly 10^6^ near the contrast match point (black solid curve).

Under these contrast matching conditions, phase separation into L_d_ (*ρ*_L_d__ = 0.04 fm Å^–3^) and L_o_ (*ρ*_L_o__ = 0.32 fm Å^–3^) domains—respectively depleted and enriched in chain-perdeuterated saturated lipid—results in a dramatic increase in scattered intensity (colored solid curves), compared to a uniformly mixed vesicle (black solid curve). Now, a distinct peak is evident in the low-*q* regime (*q* < 0.1 Å^–1^), which steadily shifts to higher *q* upon increasing the number of domains at a fixed total domain area fraction of 0.5 (*i.e.*, decreasing the domain size, *cf.* yellow, green, and blue curves). This effect was previously observed experimentally.^102,113^ In the high-*q* regime (*q* > 0.1 Å^–1^), increased intensity or “liftoff” is observed near the minima between scattering lobes, which increases with increasing number of domains. Liftoff is typically interpreted as evidence for transbilayer asymmetry,^82,114^ but clearly can also originate from lateral SLD fluctuations, especially in SANS experiments where bilayers contain both protiated and deuterated lipids.

We conclude this section with a brief comment on isotopic labeling. It is well known that chain perdeuteration lowers the gel/fluid melting transition temperature by 2–4 °C for fully saturated lipids.^115^ However, with respect to L_d_/L_o_ coexistence in ternary and quaternary mixtures, the effect of lipid perdeuteration has not been explored to our knowledge (*i.e.*, it is unknown how the presence of perdeuterated species changes the locations of phase boundaries). In our own work with DSPC/DOPC/POPC/Chol mixtures, we find that corresponding protiated and deuterated samples exhibit remarkably similar phase behavior and domain size, as judged by fluorescence resonance energy transfer (FRET), electron spin resonance (ESR), and SANS.^102,113^ For these mixtures, there is no indication that the L_o_ phase forms more- or less-readily in perdeuterated *versus* protiated mixtures.

### 6.2 Inelastic neutron scattering

Inelastic neutron scattering offers an experimental method to probe the dynamics of lipid bilayers. As detailed in Section 3.2, there are two types of scattering from neutrons, coherent and incoherent, with coherent scattering relating to collective motions of pairs of atoms and incoherent scattering relating to the motions of individual atoms. Examples of the dynamics accessible through incoherent scattering experiments include localized motions connected to head group and acyl chain relaxations, rotation of the lipid molecule, and lateral diffusion in the plane of the bilayer.^47,48,50–53,116–118^

Coherent scattering experiments are useful for probing collective vibrational motions and slower undulations of the entire bilayer. Collective vibrational features are relatively fast motions (<1 ps) connected to density fluctuations in the plane of the bilayer.^63,119,120^ The undulation motions of the bilayer are an especially interesting application of inelastic coherent neutron scattering because these motions can be related to the bending modulus of the bilayer. This has been demonstrated in homogenous lipid bilayers^66,68,69^ and subsequently used to show how the bending modulus is affected by a number of parameters including charge density,^121^ cholesterol content,^67^ and the presence of pore forming peptides.^70^ Coherent neutron scattering can also be used to investigate the mechanical properties of phase separated lipid bilayers *in situ*. In particular, by matching the SLD of one phase (*e.g.*, L_o_) to that of the solvent, it is possible to isolate the scattering from the other phase (*e.g.*, L_d_).

### 6.3 Elastic X-ray scattering

#### 6.3.1 SAXS

**Fig. 11:**
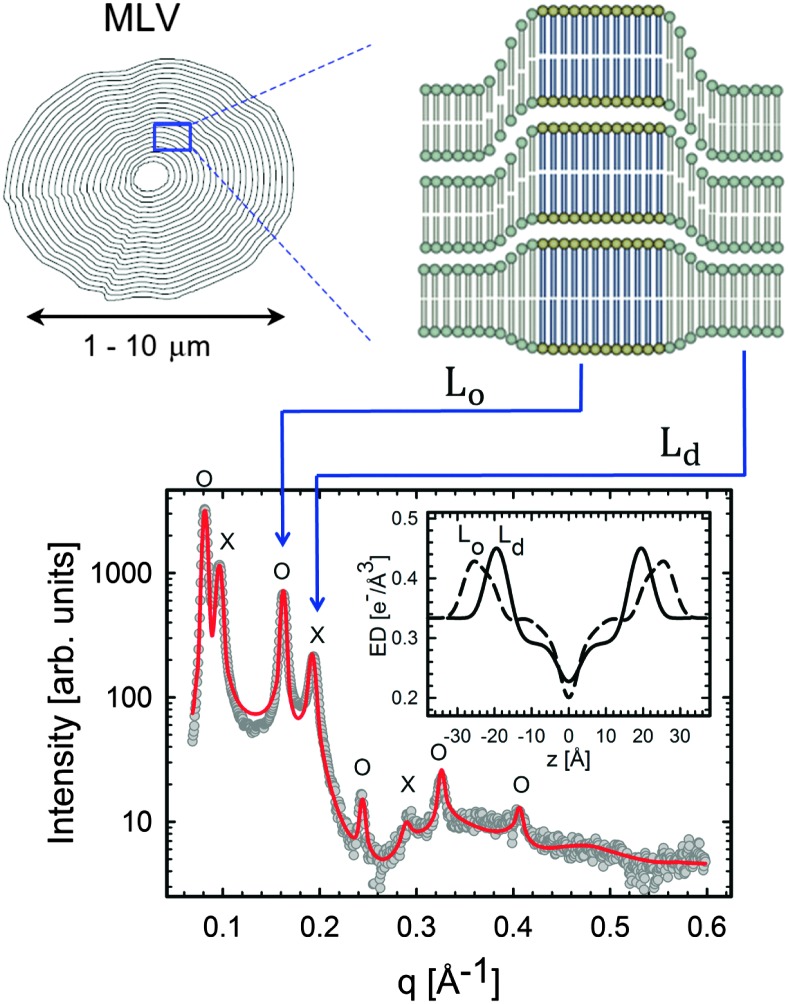
L_o_/L_d_ phase coexistence as detected by SAXS. Like domains exhibit long-range alignment and consequently display two distinct lamellar lattices. Here o's indicate peaks associated with L_o_ domains and ×'s peaks associated with L_d_ domains. The inset to the scattering pattern of DSPC/DOPC/Chol in the phase coexistence regime shows the EDP of the two domains resulting from a global fit (red solid line). Figure taken from ref. 122.

In the case of X-rays, there is no appreciable lateral contrast between the hydrocarbon regions of coexisting phases. X-ray experiments are therefore poorly suited for the study of domain size and organization. However, X-rays are highly sensitive to electron density variations across the bilayer, and consequently to internal domain structure. Probing domain structure *in situ* is most easily accomplished using multibilayer stacks. In this sample preparation, like-domains are often in registry and can be detected as two separated lamellar lattices if *V*_coh_ < *V*_D_ (Fig. 11). This is typically the case for macroscopic domains on the order of a few μm.

Heftberger *et al.* ^100^ demonstrated that for MLVs, the scattered intensity can be modeled as:

38 *I*(*q*) = (1 – *c*_L_d__)*I*_L_o__(*q*) + *c*_L_d__*I*_L_d__(*q*),

where *c*_L_d__ accounts for the L_d_ phase fraction, and *I*_L_o__ and *I*_L_d__ are the scattered intensities of the liquid-ordered and liquid-disordered phases, respectively, and are given by eqn (21). Thus, every phase is described by a separate structure factor (eqn (7)) and form factor (eqn (17)).

**Fig. 12:**
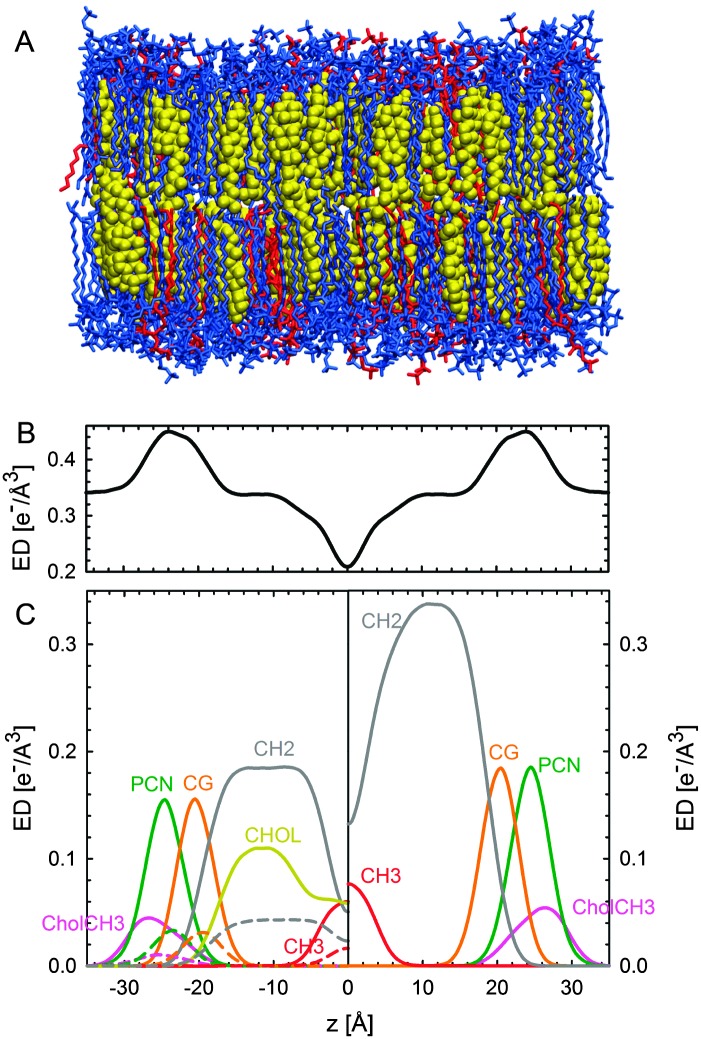
Parsing scheme of ternary lipid mixtures based on MD simulations of an L_o_ phase (panel A, DPPC lipids are drawn in blue, DOPC in red, and cholesterol in yellow). Panel B shows the electron density profile calculated from simulations, and panel C the electron densities of individual molecular groups. The left side panel shows the individual contributions of DPPC (solid lines) and DOPC (dashed lines) for the CholCH_3_, PCN, CG, CH_2_ and CH_3_ groups. The contribution of cholesterol is shown as a separate yellow line. The panel on the right shows the condensed parsing scheme after merging individual contributions. Figure taken from ref. 100 with permission.

Having established the SDP analysis for MLVs^98^ (see above), it is reasonably straightforward to extend this model to coexisting domains. However, since each domain has a characteristic lipid composition (in the case of ternary mixtures, a high-*T*_m_ lipid, a low-*T*_m_ lipid and cholesterol) the underlying parsing scheme of quasi-molecular fragments must average over the contributions of each lipid, as illustrated in Fig. 12.

In order to establish this analysis, data from tieline endpoint samples were compared with tieline midpoint samples and were found to be in good agreement (within experimental uncertainty).^100^ Results of the *in situ* study of DOPC/DPPC/Chol and DOPC/DSPC/Chol showed that L_o_ domains are about 9–10 Å thicker than L_d_ phases (consistent with SANS measurements^102^), and that their area per lipid is about 20 Å^2^ smaller (Table 2). Due to the presence of a high-*T*_m_ lipid and the condensing effect of cholesterol, L_o_ phases are considerably more rigid than L_d_ domains. Thus, their Caillé parameter is about 65% smaller and the number of Bragg peaks is almost double that of those from the L_d_ phase. Further increase to the overall cholesterol concentration decreased the differences between L_o_ and L_d_. This suggests that the L_o_ phase is saturated with cholesterol, and that additional cholesterol incorporates itself into the L_d_ phase.

**Table 2:** Structural results and bending fluctuations for coexisting L_d_/L_o_ domains.^100^ Parameter uncertainties are <2%

	*d*_B_ (Å)	*A* (Å^2^)	*η*
DOPC/DPPC/Chol^*a*^-L_d_	37.9	64.9	0.074
DOPC/DPPC/Chol^*a*^-L_o_	47.2	44.4	0.021
DOPC/DSPC/Chol^*b*^-L_d_	38.5	63.1	0.091
DOPC/DSPC/Chol^*b*^-L_o_	49.8	43.2	0.030

^*a*^Molar fractions: DOPC (0.37), DPPC (0.47), Chol (0.16), *T* = 15 °C.

^*b*^Molar fractions: DOPC (0.42), DSPC (0.37), Chol (0.21), *T* = 22 °C.

**Fig. 13:**
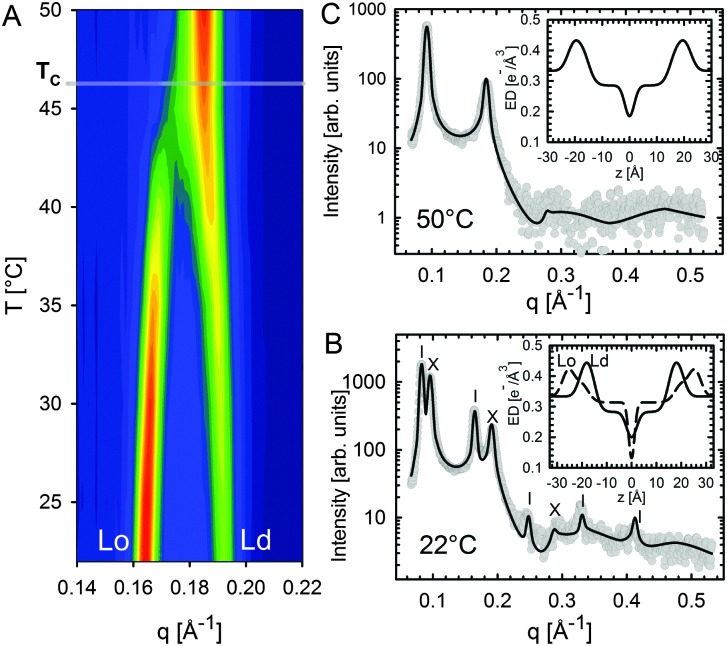
Melting of L_o_ domains in DOPC/DSPC/Chol. Panel A shows a contour plot of second order Bragg reflections associated with L_o_ and L_d_ phases. Above *T*_c_, only a single lamellar lattice is observed. Panel B shows Bragg scattering from L_o_ (dashes) and L_d_ (crosses) domains at 22 °C. Panel C is the same system at 50 °C. Best fits are shown as solid lines. Inserts to both panels show the resulting ED profiles for L_o_ and L_d_ phases. Figure taken from ref. 100 with permission.

Heftberger and co-workers^100^ additionally studied the temperature behavior of phase separated systems across the transition to a homogeneous phase (Fig. 1). In SAXS, this event is observed as a merging of the lamellar diffraction peaks (Fig. 13). Analysis of the corresponding diffraction patterns showed that melting of the L_o_ phase is associated with a decrease in bilayer thickness, and an increase in area per lipid and bending fluctuations. This is typical of fluid phase bilayers.^91,123^ In contrast, L_d_ shows the opposite behavior (*i.e.*, increased *d*_B_, and a decrease in *A* and *η*).^100^ The most likely explanation for these findings is that at temperatures below *T*_c_ cholesterol diffuses from the L_o_ to the L_d_ phase. This process is accelerated as *T*_c_ is approached from below, in agreement with a previous NMR observation.^124^

The *in situ* analysis of coexisting phases detailed above relies on long-range positional correlations of like-domains in multibilayers. Such order has been directly observed using depth-resolved confocal microscopy,^125^ and poses a challenging scientific question: “*Why are the observed domains in registry?*” The answer to this question is intimately related to the forces present between the domains, which (in the case of neutral membranes) include van der Waals, hydration, and undulation repulsion forces.^126^

That SAXS is able to differentiate between coexisting L_o_ and L_d_ domains offers the possibility to distinguish between these interactions using osmotic stress experiments. In such experiments, osmotic pressure is induced by large neutral polymers, such as polyethylene glycol.^127^ Due to their size, the polymers are excluded from the interbilayer water layer, generating osmotic pressure that decreases bilayer separation. Bilayer separation as a function of osmotic pressure is then measured using SAXS (see *e.g.* ref. 128 and 129), and the data is fitted using functional forms of the interaction potentials, in turn yielding the underlying inter-membrane forces. However, when entropically driven bending undulations are present, the standard Derjaguin–Landau–Verwey–Overbeek (DLVO) paradigm (which allows for the treatment of solvent-mediated interactions) is, strictly speaking, not applicable.^126^ Instead, a mean-field/additivity approximation can be employed, where conformational fluctuation effects on the bare interaction potentials are included in a self-consistent manner.^130–133^ Moreover, through measurements of the Caillé parameter, the mean square fluctuations of the bilayer separation,39
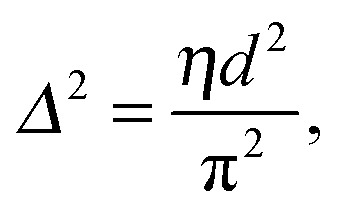
can be derived as a function of osmotic pressure by SAXS, allowing one to separate fluctuation contributions from bare interactions.^134^

**Fig. 14:**
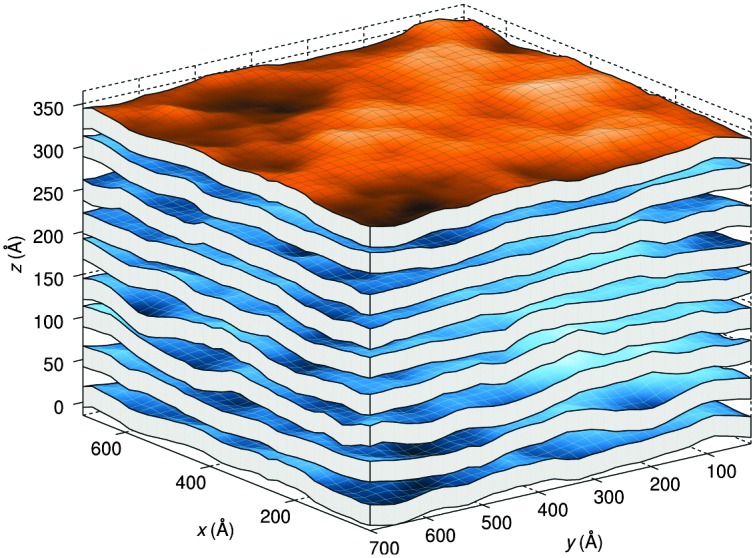
Real-space snapshots of equilibrium L_d_ simulations at a given osmotic pressure. Figure taken from ref. 137 with permission.

**Fig. 15:**
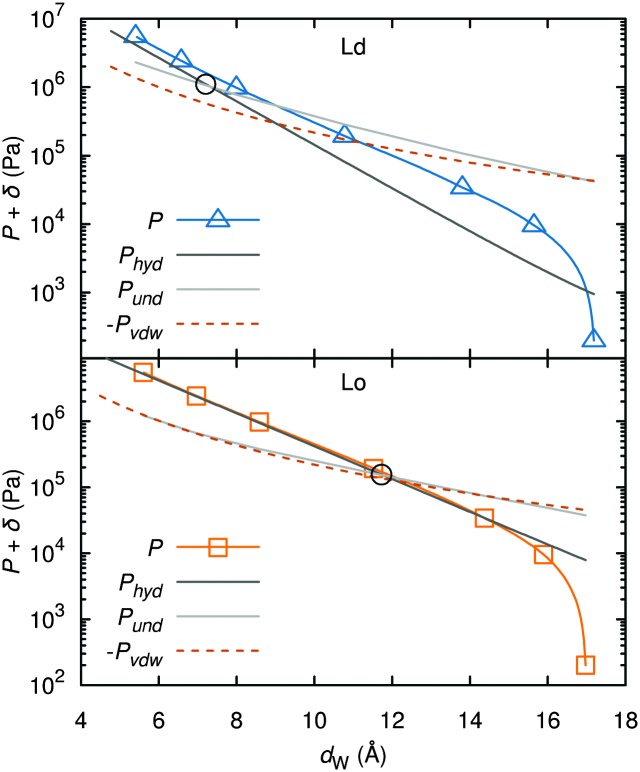
Deconstruction of the total osmotic pressure, *P*, into contributions of hydration, *P*_hyd_, van der Waals, *P*_vdW_, and undulation interactions, *P*_und_, for coexisting L_d_ (upper) and L_o_ (lower) domains. Open black circles show the *d*_W_ values at which the hydration and undulation pressures are equal. Figure taken from ref. 137 with permission.

A different approach from the above is Monte Carlo (MC) simulations.^135,136^ Recently, Kollmitzer *et al.* ^137^ explored this approach for coexisting L_o_/L_d_ domains, by coupling MC simulations (Fig. 14) with an optimization routine that jointly fits osmotic pressure dependencies of *d*_W_ and *Δ*. This allowed for the disentanglement of the different force contributions. Results (Fig. 14) for this analysis show only small differences in the van der Waals interactions between L_o_ and L_d_. However, the other two interactions differed significantly. L_o_ phases show a rapid decay of undulation repulsion (*i.e.*, reduced fluctuations compared to L_d_ phases), but a much slower decay in hydration repulsion. It is therefore clear that in the case of L_d_ domains, fluctuation forces dominate domain interactions over a broad range of distances, while hydration forces are most prominent in the L_o_ phase. Thus, there seems to be a delicate balance between hydration and fluctuation interactions which underlies domain alignment, an observation that should be considered in future theoretical treatments (Fig. 15).

A further benefit of the above analysis is that the domain bilayer bending rigidity, *K*_c_, can be derived from the fluctuation contributions. This is an important parameter with regard to the partitioning of proteins in either L_o_ or L_d_ domains.^138,139^ For DOPC/DSPC/Chol, Kollmitzer and co-workers^137^ reported *K*_c_ = 120 zJ for L_o_ and 44 zJ for L_d_ domains. In other words, L_d_ domains are about three times softer than L_o_ domains.

#### 6.3.2 WAXS

**Fig. 16:**
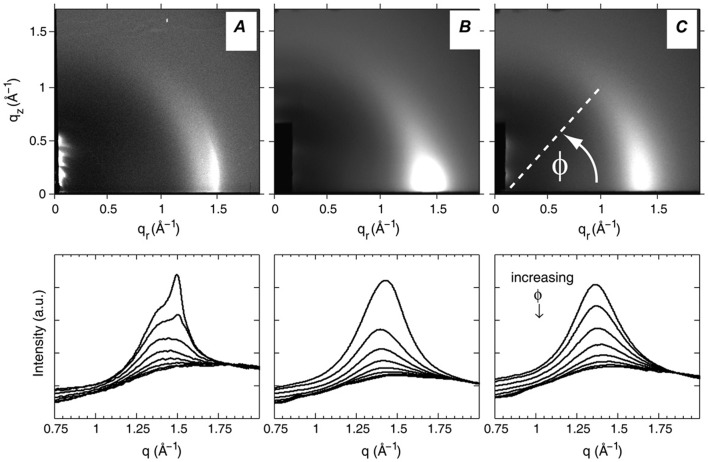
WAXS scattering from: (A) 1 : 1 DOPC/DPPC; and (B and C) 1 : 1 DOPC/DPPC/Chol (15 mol%), *T* = 25 °C and 45 °C (*T*_c_ ≃ 30 °C). The bottom row shows the corresponding *I*(*q*) plots with different *φ*-ranges (*φ* is the angle measured from the in-plane axis on the detector). Figure taken from ref. 142 with permission.

Wide-angle X-ray scattering (WAXS) reports on chain-chain positional correlations: specifically, the peak position reflects the average distance between chains, while peak width is inversely related to in-plane positional correlations. The condensing effect of cholesterol in fluid bilayers shifts and broadens the WAXS peaks of PC bilayers, as compared to the gel phase. However, even at high cholesterol concentrations (>30 mol%), the membranes still resemble fluid bilayers.^140^ Nevertheless phase coexistence may be present even when a single lamellar phase is seen in SAXS (*e.g.*, if both phases have the same *d* spacings, or if *V*X-raycho ≥ *D*). WAXS from oriented samples offers distinct advantages for examining phase separation. In such systems, off-axis scattering intensity is related to the distribution of acyl chain tilt angles, and the width of this distribution gives rise to an X-ray order parameter^141,142^
40
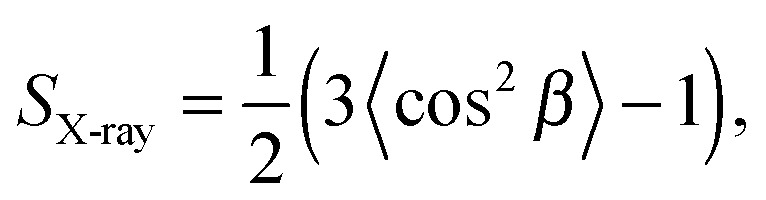
where *β* is the average tilt angle. *S*_X-ray_ is markedly different for L_d_ and L_o_ phases. It should be pointed out that the absolute magnitude of *S*_X-ray_ is different from the NMR carbon-deuterium order parameter *S*_CD_ obtained from NMR.^141^ Mills and coworkers^142^ applied this analysis to DOPC/DPPC/Chol mixtures (Fig. 16). In the phase coexistence regime they found that two tilt distributions were required to model the data (Fig. 16B), resulting in *S*_X-ray_ ∼ 0.7 for L_o_ and *S*_X-ray_ ∼ 0.4 for L_d_ domains, while only a single order parameter was needed at temperatures *T* > *T*_c_ and for binary DOPC/DPPC mixtures (Fig. 16A and C).

## 7 Conclusions

Over the past 50 years neutron and X-ray scattering have contributed significantly to our knowledge of lipid membrane structure. With the advent of full *q*-range models – culminating in the SDP model – high-resolution structural information has been obtained. More recently, ULVs have been extensively used to interrogate phase separated systems, enabling new approaches for the study of static and dynamic structures. Importantly, inelastic scattering has developed to the point where it is possible to measure, *in situ*, the mechanical properties of nanoscopic domains populating ULVs. SANS on similar samples has provided unprecedented resolution of static domain structure and how domain size correlates with bilayer thickness mismatch between L_o_ and L_d_ domains.^102^ Recently, the effect of cholesterol and temperature on domain structure and bilayer elasticity,^122,143^ as well as inter-domain forces,^137^ have provided us with further insights into mechanisms that stabilize domains.

It is hoped that future studies will explore questions including: the effect of membrane proteins on domains; ion-specific interactions; and the effect of bilayer asymmetry on domain structure and dynamics. In particular, bilayer asymmetry may change our current views on the role of lipids in plasma membranes.^71,144^ Ultimately, all of these efforts will fully be put to use to study the static and dynamic^145^ structure of live cells.

## References

[cit1] Lingwood D., Simons K. (2010). Science.

[cit2] Kusumi A., Fujiwara T. K., Chadda R., Xie M., Tsunoyama T. A., Kalay Z., Kasai R. S., Suzuki K. G. N. (2012). Annu. Rev. Cell Dev. Biol..

[cit3] Kraft M. L. (2013). Mol. Biol. Cell.

[cit4] Sevcsik E., Brameshuber M., Fölser M., Weghuber J., Honigmann A., Schütz G. J. (2015). Nat. Commun..

[cit5] Feigenson G. W. (2009). Biochim. Biophys. Acta.

[cit6] Marsh D. (2009). Biochim. Biophys. Acta.

[cit7] Heberle F. A., Petruzielo R. S., Goh S. L., Konyakhina T. M., Ackermann D. G., Amazon J. J. and Feigenson G. W., in Liposomes, Lipid Bilayers and Model Membranes, ed. G. Pabst, N. Kučerka, M.-P. Nieh and J. Katsaras, CRC Press, Boca Raton, FL, 2014, pp. 143–165.

[cit8] Pabst G., Kučerka N., Nieh M.-P., Rheinstädter M. C., Katsaras J. (2010). Chem. Phys. Lipids.

[cit9] Hill L. T., An Introduction to Statistical Thermodynamics, Dover Publications, New York, NY, 1986.

[cit10] Heimburg T., Thermal Biophysics of Membranes, Wiley-VCH Verlag GmbH & Co. KGaA, Weinheim, Germany, 2007.

[cit11] Brüning B., Wald E., Schrader W., Behrends R., Kaatze U. (2009). Soft Matter.

[cit12] Almeida P. F. (2009). Biochim. Biophys. Acta.

[cit13] Heberle F. A., Feigenson G. W. (2011). Cold Spring Harbor Perspect. Biol..

[cit14] Marsh D. (2010). Biochim. Biophys. Acta, Biomembr..

[cit15] Kuzmin P. I., Akimov S. A., Chizmadzhev Y. A., Zimmerberg J., Cohen F. S. (2005). Biophys. J..

[cit16] Buboltz J. T., Feigenson G. W. (1999). Biochim. Biophys. Acta.

[cit17] Huang J., Buboltz J. T., Feigenson G. W. (1999). Biochim. Biophys. Acta, Biomembr..

[cit18] Wassall S. R., Brzustowicz M. R., Shaikh S. R., Cherezov V., Caffrey M., Stillwell W. (2004). Chem. Phys. Lipids.

[cit19] Silvius J. R. (2003). Biophys. J..

[cit20] Mouritsen O. G., Jørgensen K. (1994). Chem. Phys. Lipids.

[cit21] Ayuyan A. G., Cohen F. S. (2006). Biophys. J..

[cit22] Zhao J., Wu J., Heberle F. A., Mills T. T., Klawitter P., Huang G., Costanza G., Feigenson G. W. (2007). Biochim. Biophys. Acta, Biomembr..

[cit23] Veatch S. L., Leung S. S. W., Hancock R. E. W., Thewalt J. L. (2007). J. Phys. Chem. B.

[cit24] Neutron scattering in biology: Techniques and applications, ed. J. Fitter, T. Gutberlet and J. Katsaras, Springer, Berlin and New York, 2006.

[cit25] Born M. and Wolf E., Principles of optics: Electromagnetic theory of propagation, interference and diffraction of light, Pergamon Press, Oxford and New York, 6th edn, 1980.

[cit26] Bernhoef N., Hiess A., Langridge S., Stunault A., Wermeille D., Vettier C., Landler G. H., Huth M., Jourdan M., Adrian H. (1998). Phys. Rev. Lett..

[cit27] Felber J., Gäler R., Golub R., Prechtl K. (1998). Physica B.

[cit28] Chen S. H., Liao C. Y., Huang H. W., Weiss T. M., Bellisent-Funel M. C., Sette F. (2001). Phys. Rev. Lett..

[cit29] Weiss T. M., Chen P. J., Sinn H., Alp E. E., Chen S. H., Huang H. W. (2003). Biophys. J..

[cit30] Rheinstädter M. C., in Neutron scattering in biology, ed. J. Fitter, T. Gutberlet and J. Katsaras, Springer, Berlin and New York, 2006, pp. 263–286.

[cit31] Klein R., in Neutron, X-rays and light, ed. P. Lindner and T. Zemb, North-Holland, Amsterdam, 2002, pp. 351–379.

[cit32] Jeu W. H. d., Ostrovskii B. I., Shalaginov A. N. (2003). Rev. Mod. Phys..

[cit33] Caillé A. (1972). C. R. Acad. Sci., Paris B.

[cit34] Zhang R., Suter R. M., Nagle J. F. (1994). Phys. Rev. E: Stat. Phys., Plasmas, Fluids, Relat. Interdiscip. Top..

[cit35] Pabst G., Rappolt M., Amenitsch H., Laggner P. (2000). Phys. Rev. E: Stat. Phys., Plasmas, Fluids, Relat. Interdiscip. Top..

[cit36] Reusch T., Schülein F. J. R., Nicolas J. D., Osterhoff M., Beerlink A., Krenner H. J., Müller M., Wixforth A., Salditt T. (2014). Phys. Rev. Lett..

[cit37] Brockhouse B. N., Stewart A. T. (1955). Phys. Rev..

[cit38] Copley J., Udovic T. (1993). J. Res. Natl. Bur. Stand..

[cit39] Maier-Leibnitz H. (1966). Nukleonik.

[cit40] Mezei F. (1972). Z. Phys..

[cit41] Van Hove L. (1954). Phys. Rev..

[cit42] Bée M., Quasielastic neutron scattering: Principles and applications in solid state chemistry, biology, and materials science, Adam Hilger, Bristol, England and Philadelphia, 1988.

[cit43] Gennes P. G. d. (1963). Solid State Commun..

[cit44] Zaccai G. (2000). Science.

[cit45] Pfeiffer W., Henkel T., Sackmann E., Knoll W., Richter D. (1989). Europhys. Lett..

[cit46] Wood K., Frölich A., Paciaroni A., Moulin M., Härtlein M., Zaccai G., Tobias D. J., Weik M. (2008). J. Am. Chem. Soc..

[cit47] Armstrong C. L., Kaye M. D., Zamponi M., Mamontov E., Tyagi M., Jenkins T., Rheinstädter M. C. (2010). Soft Matter.

[cit48] König S., Pfeiffer W., Bayerl T., Richter D., Sackmann E. (1992). J. Phys. II.

[cit49] Rheinstädter M., in Dynamics of Soft Matter, ed. V. García Sakai, C. Alba-Simionesco and S.-H. Chen, Springer US, Boston, MA, 2012, pp. 263–286.

[cit50] Rheinstädter M., Seydel T., Demmel F., Salditt T. (2005). Phys. Rev. E: Stat., Nonlinear, Soft Matter Phys..

[cit51] Swenson J., Kargl F., Berntsen P., Svanberg C. (2008). J. Chem. Phys..

[cit52] Fitter J., Lechner R. E., Dencher N. A. (1999). J. Phys. Chem. B.

[cit53] König S., Sackmann E., Richter D., Zorn R., Carlile C., Bayerl T. M. (1994). J. Chem. Phys..

[cit54] Nickels J. D., O'Neill H., Hong L., Tyagi M., Ehlers G., Weiss K. L., Zhang Q., Yi Z., Mamontov E., Smith J. C., Sokolov A. P. (2012). Biophys. J..

[cit55] Vineyard G. H. (1958). Phys. Rev..

[cit56] Fitter G. B. J., Lechner R. E., Dencher N. A. (1996). Proc. Natl. Acad. Sci. U. S. A..

[cit57] Copley J. R. D., Rowe J. M. (1974). Phys. Rev. Lett..

[cit58] Nielsen M. (1973). Phys. Rev. B: Solid State.

[cit59] Buchenau U., Prager M., Nücker N., Dianoux A. J., Ahmad N., Phillips W. A. (1986). Phys. Rev. B: Condens. Matter Mater. Phys..

[cit60] Carpenter J. M., Price D. L. (1985). Phys. Rev. Lett..

[cit61] Nickels J. D., Perticaroli S., O'Neill H., Zhang Q., Ehlers G., Sokolov A. P. (2013). Biophys. J..

[cit62] Neumann D. A., Copley J. R. D., Cappelletti R. L., Kamitakahara W. A., Lindstrom R. M., Creegan K. M., Cox D. M., Romanow W. J., Coustel N., McCauley J. P., Maliszewskyj N. C., Fischer J. E., Smith A. B. (1991). Phys. Rev. Lett..

[cit63] Rheinstädter M. C., Ollinger C., Fragneto G., Demmel F., Salditt T. (2004). Phys. Rev. Lett..

[cit64] Buchenau U., Wischnewski A., Richter D., Frick B. (1996). Phys. Rev. Lett..

[cit65] Engberg D., Wischnewski A., Buchenau U., Börjesson L., Dianoux A. J., Sokolov A. P., Torell L. M. (1998). Phys. Rev. B: Condens. Matter Mater. Phys..

[cit66] Woodka A. C., Butler P. D., Porcar L., Farago B., Nagao M. (2012). Phys. Rev. Lett..

[cit67] Arriaga L. R., Rodríguez-García R., López-Montero I., Farago B., Hellweg T., Monroy F. (2010). Eur. Phys. J. E: Soft Matter Biol. Phys..

[cit68] Pan J., Cheng X., Sharp M., Ho C.-S., Khadka N., Katsaras J. (2015). Soft Matter.

[cit69] Yi Z., Nagao M., Bossev D. P. (2009). J. Phys.: Condens. Matter.

[cit70] Lee J.-H., Choi S.-M., Doe C., Faraone A., Pincus P. A., Kline S. R. (2010). Phys. Rev. Lett..

[cit71] Nickels J., Smith J., Cheng X. (2015). Chem. Phys. Lipids.

[cit72] Watson M. C., Brown F. L. (2010). Biophys. J..

[cit73] Zilman A. G., Granek R. (1996). Phys. Rev. Lett..

[cit74] Katsaras J. (1997). Biophys. J..

[cit75] Katsaras J. (1998). Biophys. J..

[cit76] Armstrong C. L., Marquardt D., Dies H., Kučerka N., Yamani Z., Harroun T. A., Katsaras J., Shi A.-C., Rheinstaedter M. C. (2013). PLoS One.

[cit77] Naumann C. A., Prucker O., Lehmann T., Rühe J., Knoll W., Frank C. W. (2002). Biomacromolecules.

[cit78] Liposomes, Lipid Bilayers and Model Membranes, ed. G. Pabst, N. Kučerka, M.-P. Nieh and J. Katsaras, CRC Press, Boca Raton, FL, 2014, pp. 199–216.

[cit79] Kučerka N., Nagle J. F., Feller S. E., Balgavý P. (2004). Phys. Rev. E: Stat., Nonlinear, Soft Matter Phys..

[cit80] Kiselev M. A., Zemlyanaya E. V., Aswal V. K., Neubert R. H. H. (2006). Eur. Biophys. J..

[cit81] Pencer J., Krueger S., Adams C. P., Katsaras J. (2006). J. Appl. Crystallogr..

[cit82] Brzustowicz M. R., Brunger A. T. (2005). J. Appl. Crystallogr..

[cit83] Oliveira C. L. P., Gerbelli B. B., Silva E. R. T., Nallet F., Navailles L., Oliveira E. A., Pedersen J. S. (2012). J. Appl. Crystallogr..

[cit84] King G. I., White S. H. (1986). Biophys. J..

[cit85] Wiener M. C., King G. I., White S. H. (1991). Biophys. J..

[cit86] Wiener M. C., White S. H. (1991). Biophys. J..

[cit87] Wiener M. C., White S. H. (1991). Biophys. J..

[cit88] Wiener M. C., White S. H. (1992). Biophys. J..

[cit89] Wiener M. C., White S. H. (1992). Biophys. J..

[cit90] Kučerka N., Nagle J. F., Sachs J. N., Feller S. E., Pencer J., Jackson A., Katsaras J. (2008). Biophys. J..

[cit91] Kučerka N., Nieh M. P., Katsaras J. (2011). Biochim. Biophys. Acta.

[cit92] Kučerka N., Gallová J., Uhríková D., Balgavý P., Bulacu M., Marrink S.-J., Katsaras J. (2009). Biophys. J..

[cit93] Pan J., Marquardt D., Heberle F. A., Kučerka N., Katsaras J. (2014). Biochim. Biophys. Acta, Biomembr..

[cit94] Pan J., Cheng X., Monticelli L., Heberle F. A., Kučerka N., Tieleman D. P., Katsaras J. (2014). Soft Matter.

[cit95] Kučerka N., van Oosten B., Pan J., Heberle F. A., Harroun T. A., Katsaras J. (2015). J. Phys. Chem. B.

[cit96] Heberle F. A., Pan J., Standaert R. F., Drazba P., Kučerka N., Katsaras J. (2012). Eur. Biophys. J..

[cit97] Marsh D. (2010). Chem. Phys. Lipids.

[cit98] Heftberger P., Kollmitzer B., Heberle F. A., Pan J., Rappolt M., Amenitsch H., Kučerka N., Katsaras J., Pabst G. (2014). J. Appl. Crystallogr..

[cit99] Fogarty J. C., Arjunwadkar M., Sagar A. P., Pan J. (2015). Biochim. Biophys. Acta, Biomembr..

[cit100] Heftberger P., PhD thesis, Graz University of Technology, Austria, Graz, 2015.

[cit101] Armstrong C. L., Barrett M. A., Hiess A., Salditt T., Katsaras J., Shi A.-C., Rheinstaedter M. C. (2012). Eur. Biophys. J. Biophys. Lett..

[cit102] Heberle F. A., Petruzielo R. S., Pan J., Drazba P., Kučerka N., Standaert R. F., Feigenson G. W., Katsaras J. (2013). J. Am. Chem. Soc..

[cit103] Pencer J., Mills T. T., Kucerka N., Nieh M. P., Katsaras J. (2007). Methods Mol. Biol..

[cit104] Tristram-Nagle S., Liu Y., Legleiter J., Nagle J. F. (2002). Biophys. J..

[cit105] Tristram-Nagle S., Petrache H. I., Nagle J. F. (1998). Biophys. J..

[cit106] Greenwood A. I., Tristram-Nagle S., Nagle J. F. (2006). Chem. Phys. Lipids.

[cit107] Heberle F. A., Wu J., Goh S. L., Petruzielo R. S., Feigenson G. W. (2010). Biophys. J..

[cit108] Konyakhina T. M., Wu J., Mastroianni J. D., Heberle F. A., Feigenson G. W. (2013). Biochim. Biophys. Acta, Biomembr..

[cit109] Petruzielo R. S., Heberle F. A., Drazba P., Katsaras J., Feigenson G. W. (2013). Biochim. Biophys. Acta, Biomembr..

[cit110] Anghel V. N. P., Kučerka N., Pencer J., Katsaras J. (2007). J. Appl. Crystallogr..

[cit111] Pencer J., Mills T., Anghel V., Krueger S., Epand R. M., Katsaras J. (2005). Eur. Phys. J. E: Soft Matter Biol. Phys..

[cit112] Heberle F. A., Anghel V. N., Katsaras J. (2015). J. Appl. Crystallogr..

[cit113] Heberle F. A., Doktorova M., Goh S. L., Standaert R. F., Katsaras J., Feigenson G. W. (2013). J. Am. Chem. Soc..

[cit114] Kučerka N., Pencer J., Sachs J. N., Nagle J. F., Katsaras J. (2007). Langmuir.

[cit115] Katsaras J., Epand R. F., Epand R. M. (1997). Phys. Rev. E: Stat. Phys., Plasmas, Fluids, Relat. Interdiscip. Top..

[cit116] König S., Bayerl T. M., Coddens G., Richter D., Sackmann E. (1995). Biophys. J..

[cit117] Armstrong C. L., Häussler W., Seydel T., Katsaras J., Rheinstädter M. C. (2014). Soft Matter.

[cit118] Sharma V. K., Mamontov E., Anunciado D. B., O'Neill H., Urban V. (2015). J. Phys. Chem. B.

[cit119] Hub J. S., Salditt T., Rheinstädter M. C., Groot B. L. d. (2007). Biophys. J..

[cit120] Brüning B., Rheinstädter M. C., Hiess A., Weinhausen B., Reusch T., Aeffner S., Salditt T. (2010). Eur. Phys. J. E: Soft Matter Biol. Phys..

[cit121] Brüning B., Stehle R., Falus P., Farago B. (2013). Eur. Phys. J. E: Soft Matter Biol. Phys..

[cit122] Heftberger P., Kollmitzer B., Rieder A. A., Amenitsch H., Pabst G. (2015). Biophys. J..

[cit123] Pabst G., Amenitsch H., Kharakoz D. P., Laggner P., Rappolt M. (2004). Phys. Rev. E: Stat., Nonlinear, Soft Matter Phys..

[cit124] Davis J. H., Schmidt M. L. (2014). Biophys. J..

[cit125] Tayebi L., Ma Y., Vashaee D., Chen G., Sinha S. K., Parikh A. N. (2012). Nat. Mater..

[cit126] Israelachvili J. N., Intermolecular and surface forces, Academic Press, Burlington, MA, 3rd edn, 2011.

[cit127] Parsegian V. A., Rand R. P., Fuller N. L., Rau D. C. (1986). Methods Enzymol..

[cit128] McIntosh T. J., Simon S. A. (1993). Biochemistry.

[cit129] Parsegian V. A. and Rand R. P., in Handbook of Biological Physics, ed. R. Lipowsky and E. Sackmann, Elsevier, Amterdam, 1995, pp. 643–690.

[cit130] Sornette D., Ostrowsky N. (1986). J. Chem. Phys..

[cit131] Evans E. A., Parsegian V. A. (1986). Proc. Natl. Acad. Sci. U. S. A..

[cit132] Podgornik R., Parsegian V. A. (1992). Langmuir.

[cit133] Mecke K. R., Charitat T., Graner F. (2003). Langmuir.

[cit134] Petrache H. I., Gouliaev N., Tristram-Nagle S., Zhang R. T., Suter R. M., Nagle J. F. (1998). Phys. Rev. E: Stat. Phys., Plasmas, Fluids, Relat. Interdiscip. Top..

[cit135] Gouliaev N., Nagle J. F. (1998). Phys. Rev. E: Stat. Phys., Plasmas, Fluids, Relat. Interdiscip. Top..

[cit136] Gouliaev N., Nagle J. F. (1998). Phys. Rev. Lett..

[cit137] Kollmitzer B., Heftberger P., Podgornik R., Nagle J. F., Pabst G. (2015). Biophys. J..

[cit138] Marsh D. (2007). Biophys. J..

[cit139] Marsh D. (2008). Biophys. J..

[cit140] Engelman D. M., Rothman J. E. (1972). J. Biol. Chem..

[cit141] Mills T. T., Tristram-Nagle S., Heberle F. A., Morales N. F., Zhao J., Wu J., Toombes G. E., Nagle J. F., Feigenson G. W. (2008). Biophys. J..

[cit142] Mills T. T., Toombes G. E., Tristram-Nagle S., Smilgies D. M., Feigenson G. W., Nagle J. F. (2008). Biophys. J..

[cit143] Brüning B.-A., Prévost S., Stehle R., Steitz R., Falus P., Farago B., Hellweg T. (2014). Biochim. Biophys. Acta.

[cit144] Marquardt D., Geier B., Pabst G. (2015). Membranes.

[cit145] Nickels J., Ohl M., Cheng X., Stanley C., Heberle F., Standaert R. F., Katsaras J. Biophys. J..

